# Large extrachromosomal replicons are widespread across bacterial lineages and show coordinated replication termination and spatial coupling with the chromosome

**DOI:** 10.1038/s41467-026-72671-7

**Published:** 2026-05-02

**Authors:** Jakub Czarnecki, Morgan Lamberioux, Ole Skovgaard, Amaury Bignaud, Najwa Taib, Théophile Niault, Jasmin Ostermayer, Pascale Bourhy, Julia Bos, Elvira Krakowska, Dariusz Bartosik, Romain Koszul, Martial Marbouty, Didier Mazel, Marie-Eve Val

**Affiliations:** 1 Department of Genomes and Genetics, Bacterial Genome Plasticity Unit, Institut Pasteur, Université Paris Cité, CNRS UMR3525, Paris, France; 2 Doctoral School, Sorbonne Université, Paris, France; 3 Department of Science and Environment, Systems and Models, Roskilde University, Roskilde, Denmark; 4 Department of Genomes and Genetics, Spatial Regulation of Genomes Unit, Institut Pasteur, Université Paris Cité, CNRS UMR3525, Paris, France; 5 Institut Pasteur, Hub Bioinformatics and Biostatistics, Paris, France; 6 Biology of Spirochetes Unit, Institut Pasteur, National Reference Center for Leptospirosis, Paris, France; 7 Department of Bacterial Genetics, University of Warsaw, Faculty of Biology, Institute of Microbiology, Warsaw, Poland

**Keywords:** Bacterial genetics, Origin firing, Bacterial genomics

## Abstract

Bacterial genomes frequently harbor extrachromosomal replicons (ERs) that promote genome plasticity and adaptation, ranging from small plasmids to chromosome-scale replicons. In a few model organisms, including *Vibrio cholerae* and *Agrobacterium tumefaciens*, large ERs are coordinated with chromosome replication and cell-cycle organization by specific molecular mechanisms. Whether this applies broadly across bacteria remains unknown. Here, we analyzed more than 40,000 complete bacterial genomes to update the distribution of ERs across bacterial taxa. Their GC content converged toward that of the chromosome with increasing ER size, revealing a size-dependent trend toward chromosomal composition. Such large ERs were found as conserved genomic features in many distinct genera, consistent with independent acquisition and long-term maintenance. We selected representative strains from these lineages, spanning five taxonomic classes across three bacterial phyla, to investigate replication dynamics and spatial organization. Marker frequency analysis showed that these large ERs are maintained at the same copy number as the chromosome and often complete replication synchronously. Chromosome conformation capture further revealed frequent ER–chromosome contacts, including origin–origin interactions and extended contacts along replicated arms. Together, this exploratory study lays the groundwork for uncovering new mechanisms coordinating large-ER maintenance with the bacterial chromosome.

## Abstract (web summary)

Bacterial genomes often include extrachromosomal replicons, ranging from small plasmids to chromosome-scale replicons. Here, Czarnecki et al. analyse more than 40,000 complete bacterial genomes and show that large extrachromosomal replicons are widespread, independently established, and often coupled to chromosome replication and spatial organization.

## Introduction

Extrachromosomal replicons (ERs) allow bacteria to rapidly acquire novel traits by introducing large blocks of genes that reshape physiology, behavior, or ecological range. These replicons vary widely in size, GC content, copy number, and in their interaction with host cellular processes. While some ERs remain mobile and loosely connected to host systems, others persist long enough to be tamed, aligning their GC content with that of the host chromosome, integrating into genome maintenance mechanisms, and often expanding in size, in a process referred to as domestication^1–4^. Many ERs are conserved within genera and even across families, underscoring their evolutionary success. Such genomes are considered multipartite, consisting of the chromosome and one or more ERs that are essential for survival and competitiveness in their natural ecological context, and maintained as part of the core genome. Yet how such replicons are stabilized and integrated into the cell’s tightly regulated cycle of replication, segregation, and division^5^, which represents a key step in their domestication, remains unclear. Here, we investigate whether ERs, particularly larger ones, show evidence of functional interplay with the chromosome.

Small ERs, commonly known as plasmids, are the most widespread and variable ERs among bacteria. Typically non-essential, plasmids can be lost by their host without affecting viability. They are often viewed as molecular parasites due to their potential to impose an energetic burden on their hosts^6–8^. However, plasmids also play a significant role in host fitness by carrying adaptive genes, such as those conferring antibiotic resistance or niche-specific traits, and by increasing the genomic flexibility of their hosts without disturbing the chromosome synteny^8,9^.

To ensure stable vertical inheritance, plasmids typically rely on one of two strategies: maintaining a high copy number^10–12^, or, when they exceed a critical size, encoding dedicated partitioning systems^13,14^. In addition, plasmids are often mobile and capable of horizontal transfer via conjugation^15^, a process that further enhances their persistence within bacterial populations^16^.

Through genome shuffling and DNA acquisition, plasmids can evolve into larger replicons, giving rise to megaplasmids and chromids^1,2,17^. This transition is accompanied by a reduction in copy number, consistent with constraints on the total plasmid DNA load carried by the host cell^12^. Both megaplasmids and chromids can rely on host genome maintenance systems to ensure stable inheritance^4^. From this shared constraint, however, megaplasmids and chromids diverge in their degree of interaction with the host. Megaplasmids, although not essential, often carry genetic information that enhances survival in specific ecological niches^17^. By retaining their mobility, megaplasmids can spread horizontally through conjugation, which likely accounts for their lack of conservation even within the same species^1,2^. In contrast, chromids carry core essential genes^1,2,18,19^. They lose mobility and rely exclusively on vertical transmission^15^. This deeper integration into host genome maintenance confers chromosome-like stability, resulting in strong conservation within evolutionary lineages, often at the genus level^1,2^.

Classifying bacterial replicons remains difficult despite several proposed frameworks^1,2,17^. Most schemes define the chromosome as the DnaA-dependent replicon, typically the largest, and classify all additional replicons as ERs, encompassing plasmids, megaplasmids, chromids, and secondary chromosomes^1,2^. ER subtypes are usually distinguished using pragmatic proxies such as size, GC content, mobility, and the presence of essential core genes^1,2,17^. However, these criteria often depend on arbitrary thresholds (e.g., the ~350 kb boundary between plasmids and megaplasmids^2,17^) and on context-dependent notions of essentiality^20,21^, which can blur category boundaries and hinder consistent comparisons across taxa. This problem is exacerbated by inconsistent annotation practices in public databases: in RefSeq submissions, replicon nomenclature is assigned by submitters without standardized guidelines, so most ERs are labeled “plasmid,” only a minority “megaplasmid,” and some large ERs “secondary chromosome,” reflecting historical conventions more than explicit criteria. As a result, nomenclature alone is an unreliable basis for comparative analyses of multipartite genomes. Nevertheless, early genome-wide surveys suggested that large, conserved ERs are concentrated in specific bacterial lineages^1,2^. The current abundance of complete genomes now enables this distribution to be reassessed at much higher resolution.

Sequencing-based approaches such as Marker Frequency Analysis (MFA)^22^ and Chromosome Conformation Capture (Hi-C)^23^ have provided key insights into the maintenance of large ERs and their integration into chromosome-centered cell-cycle processes. Across several multipartite-genome models, MFA indicates that large ERs replicate with chromosome-like dynamics and often reach replication termination synchronously with the chromosome, consistent with coordinated replication timing^24–29^. Hi-C analyses further support inter-replicon coordination by revealing physical contacts between ERs and chromosomal loci, suggesting structured spatial organization. In *Vibrio cholerae*, the origin of the secondary chromosome (Chr2; *ori2*) interacts with the *crtS* site on the primary chromosome (Chr1); replication of *crtS* triggers Chr2 initiation and promotes termination synchrony^24,30^. Contacts between the replication terminus (*ter*) regions of Chr1 and Chr2 have also been observed and may arise from shared subcellular positioning of *ter* regions, potentially linked to coordinated segregation at cell division^24,31,32^. In *Agrobacterium tumefaciens*, Hi-C similarly revealed interactions among the replication origins of all replicons, including the circular primary chromosome, the linear secondary chromosome, and the two large plasmids (pAt and pTi)^28^. These contacts reflect shared subcellular positioning of origin regions, driven by coordinated partitioning^33^. Together, these case studies suggest that replication coordination and spatial association can accompany large-ER domestication, but it remains unclear how general these features are across bacterial diversity, as most evidence comes from a limited set of model lineages.

In this study, we combine large-scale comparative genomics with MFA and Hi-C to explore and analyze ERs replications and interactions with main chromosome in various bacterial lineages. We first perform an agnostic census of ER diversity across ~40,000 complete bacterial genomes to identify lineages in which large ERs are recurrent and lineage-associated. We then apply MFA and Hi-C to representative strains carrying large ERs to assess their copy-number patterns, coordinated replication dynamics, and spatial association with the chromosome, providing evidence that these features recur across multiple lineages.

## Results

### Chromosomes and ERs occupy distinct GC–size space, with limited overlap

We analyzed 43,074 complete bacterial genomes from RefSeq, spanning 1955 genera and 52 phyla (Supplementary Data 1, Associated Data 1 available at the GitHub repository https://meveval-ip.github.io/ER-distribution-MFA/).

**Fig. 1:**
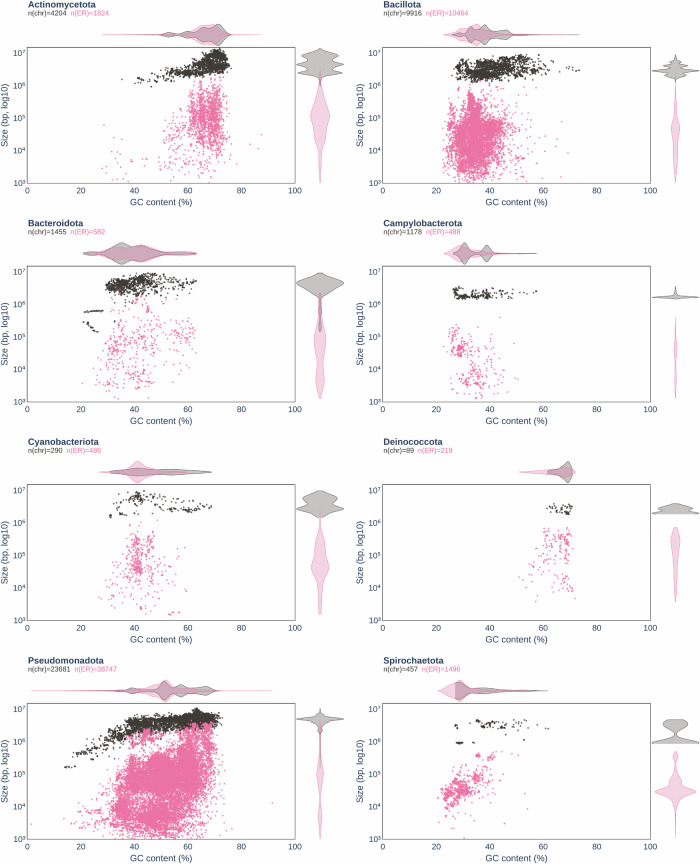
GC content (%) and size (bp, log10 scale) of bacterial replicons across major phyla. Scatter plots show all bacterial replicons positioned by GC content (%) and replicon size (bp, log₁₀ scale) for phyla with at least 100 ERs in the dataset. Points represent individual replicons, with chromosomes (grey) and ERs (pink). Marginal distributions are shown as violin plots based on kernel density estimates (KDEs), illustrating the distributions of GC content (top) and replicon size (right) separately for chromosomes (grey) and ERs (pink) within each phylum. KDEs are normalized to the same maximum width, allowing visual comparison of distribution shapes but not of the absolute numbers of replicons in each category. Numbers indicate the total counts of chromosomes (n(chr)) and ERs (n(ER)) per phylum. The number of chromosomes (n(chr)) corresponds to the number of genomes, as each genome contains a single chromosome. Interactive versions of all plots are available in Associated Data 2.

For consistency across this large dataset, we defined the chromosome as the largest replicon in each genome as in refs. ^1,2,11,34^ (see “ER dataset” section in “Methods”). Thus, all smaller replicons were considered ERs. We examined the distribution of chromosomes and ERs using two-dimensional plots of GC content versus replicon size. Figure 1 shows these distributions for phyla represented by at least 100 ERs. Across phyla, chromosomes and ERs occupy largely distinct size ranges, with some degree of overlap. This overlap is most pronounced in *Pseudomonadota*, a phylum enriched in large ERs, where certain ERs partially overlap with the chromosome cluster. At lower taxonomic levels (classes, orders, families, and genera), this overlap decreases markedly (Associated Data 2). Most genera display no size overlap between chromosomes and ERs, and only a few exceed 0.03 overlap: *Paraburkholderia* (0.259), *Burkholderia* (0.199), *Brucella/Ochrobactrum* (0.073), and *Rhizobium/Agrobacterium* (0.033) (Associated Data 2, Associated Data 3). A similar, though slightly less pronounced, trend is observed for GC content. In most lineages, ERs display lower GC content than chromosomes. A few groups deviate from this trend, showing higher ER GC content than chromosomes, including the phylum *Bacteroidota*, the families *Morganellaceae* and *Brucellaceae*, and the genera *Arsenophonus*, *Acetobacter*, *Providencia*, *Proteus*, and *Brucella/Ochrobactrum* (Associated Data 2).

### GC composition of large ERs converges toward that of their host chromosomes

The analysis of GC content versus replicon size shows that, when using absolute values, ERs form broad, highly dispersed clouds. Such absolute representations can obscure shared features, because ERs evolve within the compositional and genomic context of their host chromosomes, which GC content and size vary widely across bacterial lineages. As a result, ERs from distinct species may appear unrelated in absolute space even if they occupy comparable positions relative to their host chromosomes.

**Fig. 2:**
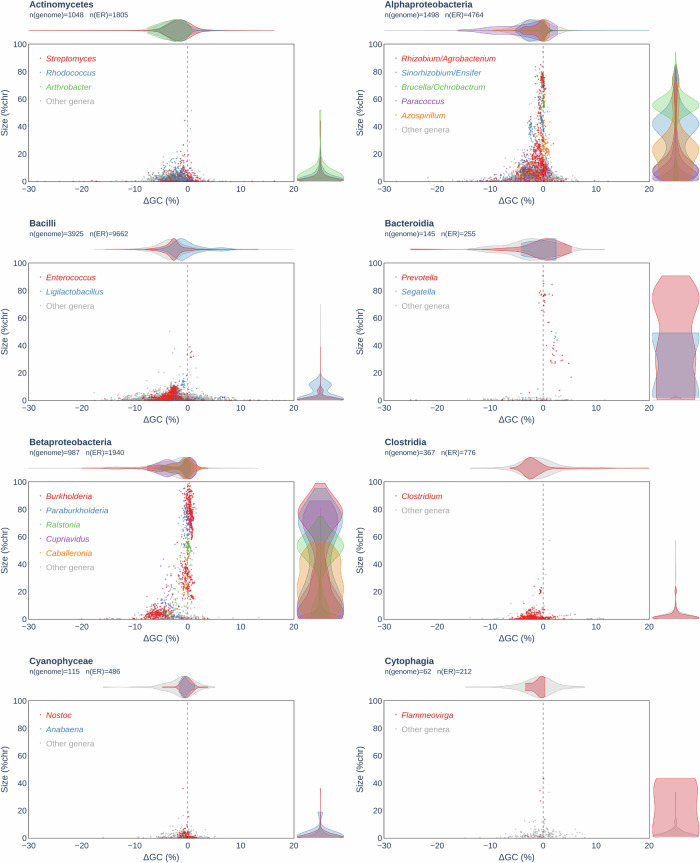
ΔGC (%) and relative size (%chr) of ERs across bacterial classes (Actinomycetes, Alphaproteobacteria, Bacilli, Bacteroidia, Betaproteobacteria, Clostridia, Cyanophyceae, Cytophagia). Scatter plots show ERs positioned by the difference in GC content relative to the chromosome of the same genome (ΔGC, %) and ER size expressed as a percentage of chromosome size (%chr) for bacterial classes with at least 100 ERs in the dataset. Points represent individual ERs. For each class, ERs belonging to selected genera are highlighted by color, while ERs from all remaining genera are shown in grey. Marginal distributions are shown as violin plots based on KDEs, illustrating the distributions of ΔGC (top) and ER size (%chr; right). KDEs are normalized to the same maximum width, allowing comparison of distribution shapes but not of the absolute numbers of ERs. A dashed lines indicates ΔGC = 0, corresponding to ERs with the same GC content as their associated chromosome. The remaining bacterial classes are shown in Fig. 3. Interactive versions of all plots are available in Associated Data 6.

**Fig. 3:**
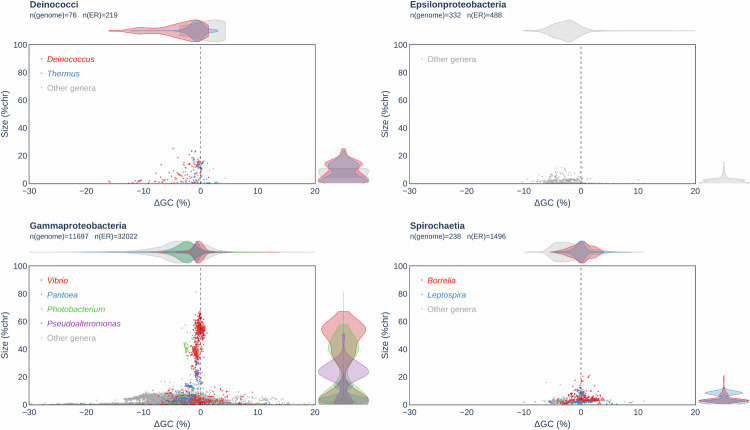
ΔGC (%) and relative size (%chr) of ERs across bacterial classes (Deinococci, Epsilonproteobacteria, Gammaproteobacteria, Spirochaetia). Same representation and analysis as in Fig. 2, shown for the remaining bacterial classes meeting the same inclusion criteria. Interactive versions of all plots are available in Associated Data 6.

To overcome this limitation, ER size and base composition were analyzed using chromosome-normalized metrics defined as ΔGC = (GC_ER_–GC_chr_) and %chr = (ER size expressed as a fraction of the host chromosome) (Associated Data 4) (see “Methods”/“Chromosome-normalized GC and size analyses”). This normalization reveals a striking and consistent trend: large ERs have a clear tendency to cluster around ΔGC ≈ 0, whereas small ERs remain broadly dispersed. Across virtually all well-sampled taxa ( ≥ 100 ERs), this trend is consistent with widespread convergence of large ERs toward host-chromosome GC content (Associated Data 5) (see “Methods”/“Statistical assessment of ΔGC–size trends”). Applying these normalized metrics across major taxonomic classes revealed consistent lineage-level patterns (Figs. 2 and 3—Interactive version Associated Data 6). ERs larger than 50%chr are particularly common in Alphaproteobacteria, Betaproteobacteria, Gammaproteobacteria, and Bacteroidia, but are rare or absent in most other classes (Associated Data 6). ΔGC distributions also vary across classes: in Actinomycetes, Bacilli, and Cyanophyceae, ΔGC values are unimodally distributed, with a peak below or around 0, whereas in Betaproteobacteria, the distribution is bimodal, with one peak near 0 and another several percentage points below chromosomal GC content (Associated Data 5). Size distributions (%chr) recapitulate these patterns: classes that rarely carry large ERs (e.g., Cytophagia) show unimodal %chr distributions dominated by small plasmids, whereas classes that include both small plasmids and large ERs (e.g., Alphaproteobacteria, Gammaproteobacteria, and Betaproteobacteria) display multimodal %chr distributions with distinct peaks corresponding to small and large replicons (Associated Data 5).

### Genus-level analysis of ER prevalence and size distributions

**Fig. 4:**
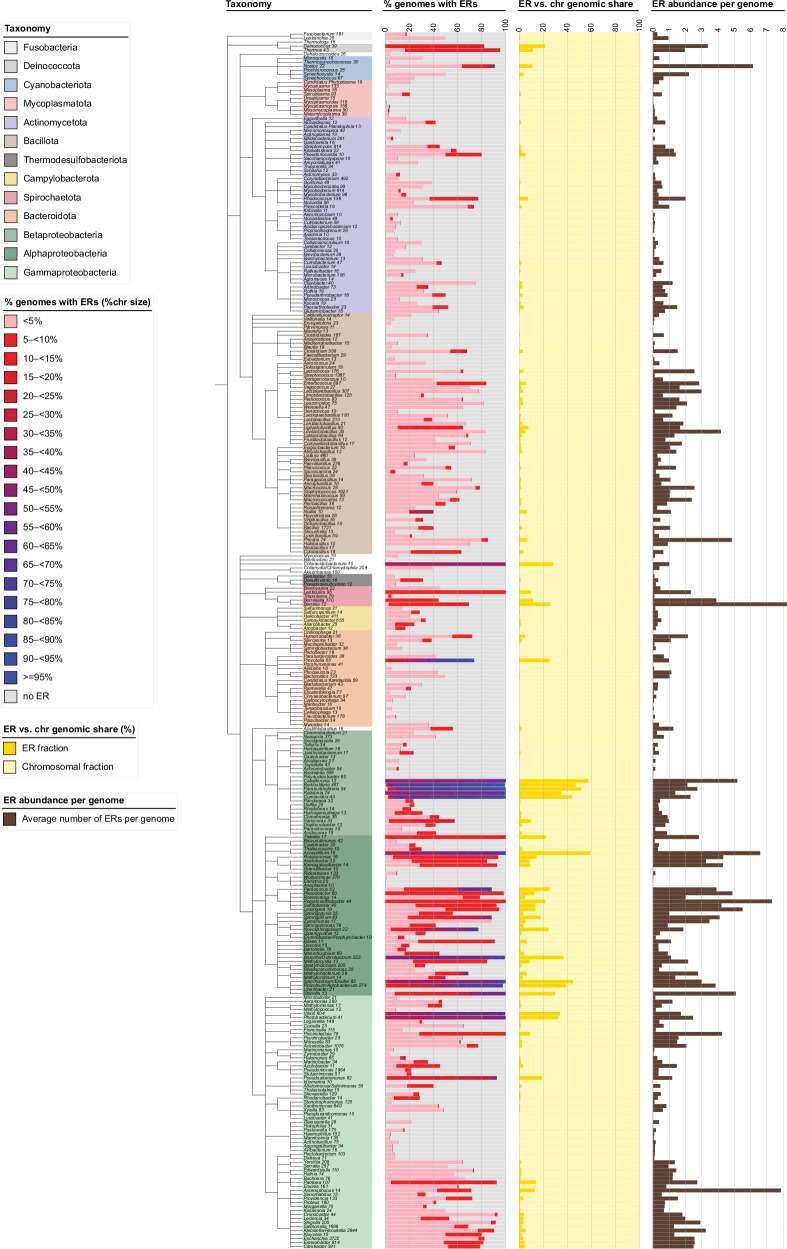
Mapping of ER distribution on a reference tree of bacteria. Genera were placed on a schematic taxonomy following the NCBITaxa classification, and only genera represented by ≥10 complete genomes in our dataset were included. For each genus, three complementary measures are shown. Left metadata panel: “% genomes with ERs” (largest-ER binning). Each genome contributes exactly one observation, defined by the size of its largest ER relative to chromosome length. Largest ER sizes were assigned to 5%chr bins (0–5%, 5–10%, …), and for each genus, we report the percentage of genomes falling into each bin. Genomes lacking ERs contribute to a dedicated “no ER” category and are shown as grey. Genomes carrying ERs are shown as colored bars: light pink indicates genera where the largest ER is <5%chr, and progressively darker red-to-blue shades indicate successive 5%chr size bins toward larger ERs. This panel, therefore, captures how frequently ERs occur within each genus and whether ER presence is dominated by small plasmids or extends into large/very large chromid-like replicons. Middle metadata panel: “ER vs. chr genomic share”. For each genus, we calculated the proportion of total genomic DNA encoded on ERs (sum of all ER lengths divided by total genome length), providing an estimate of the overall genomic contribution of ERs beyond the single-largest-replicon view. Right metadata panel: “ER abundance per genome”. For each genus, we report the mean number of ERs per genome, capturing whether genomes typically carry few replicons or are strongly plasmid-rich. Additional details are provided in Supplementary Note 1. Complementary trees at both genus and family levels are provided in Associated Data 7.

Because large, conserved ERs are often lineage-associated at the genus level^1,2^, we conducted our analysis at this taxonomic rank. Figure 4 presents a schematic genus-level phylogenetic tree annotated with ER prevalence (Associated Data 7 provides complementary tree views at both genus and family levels). We focused on 304 genera represented by ≥10 complete genomes and summarized ER distribution using three measures: (i) the percentage of genomes carrying ERs, binned by the size of the largest ER (5%chr bins), (ii) the fraction of total genomic DNA encoded on ERs, and (iii) the mean number of ERs per genome (see “Methods”/“Taxonomic bacterial tree with ER prevalence”).

Across all genomes, 22,188 of 43,074 (51.5%) did not contain any ER (grey bars in Fig. 4, “% genomes with ERs”). In contrast, 20,886 genomes (48.5%) carried at least one ER, shown in light pink for genomes whose largest ER is <5%chr, and in progressively darker red-to-blue shades for genomes whose largest ER falls into successive 5%chr size bins. At the phylum level (considering only phyla represented by ≥100 genomes), ER distribution was highly uneven. ERs were most prevalent in Pseudomonadota (59.9%), Spirochaetota (52.1%), Bacillota (43.5%), Chlamydiota (42.2%), and Cyanobacteriota (39.7%). Intermediate levels were observed in Campylobacterota (28.2%), Actinomycetota (25.2%), Thermodesulfobacteriota (22.0%), Bacteroidota (20.6%), and Fusobacteriota (23.1%). By contrast, ERs were rare in Mycoplasmatota (5.5%) and Verrucomicrobiota (2.2%). At finer taxonomic resolution, ER absence remained common. Forty-three genera represented by at least ten genomes contained no detectable ERs, although most were sparsely sampled. Among well-sampled genera (≥ 50 genomes), twenty showed ≤10% of genomes carrying ERs, including lineages with complete absence (e.g., *Candidatus* Karelsulcia and *Polynucleobacter*) and others with sporadic detection (e.g., *Bordetella*, *Wolbachia*, *Mycoplasmoides*, *Akkermansia*, *Mycoplasma*, *Haemophilus*, *Streptococcus*, *Flavobacterium*, and *Rickettsia*) (Supplementary Note 1).

### Recurrent, lineage-associated emergence of large ERs

Across bacterial genomes, ER size distributions were highly skewed: the overwhelming majority (86.0%; 47,085/54,710) were smaller than 5% of chromosome size (%chr). Among the 304 genera represented by at least ten genomes, 110 (36.2%) contained exclusively such small ERs. ERs larger than 50%chr were rarer still, comprising 1434 ERs (2.62% of all ERs), and only 23 genera (7.6%) included genomes carrying at least one. Figure 4 depicts the distribution of the sizes of the largest ERs per genome as a red–blue gradient. For a quick, genus-level overview, Supplementary Table 1 summarizes genera using arbitrary size thresholds and prevalence cutoffs.

Across the 304 genera represented in Fig. 4, ER impact is further captured by two complementary metrics: the fraction of total genomic DNA encoded on ERs and the mean number of ERs per genome. Most genera show minimal ER contribution, typically <5% of total DNA on ERs even when multiple plasmids are present. In contrast, a subset of genera devotes a substantial fraction of their genomes to ERs, largely driven by large replicons (e.g., *Azospirillum* 59.4%, *Caballeronia* 57.8%, *Paraburkholderia* 51.7%, *Sinorhizobium/Ensifer* 44.8%, *Cupriavidus* 44.2%; Fig. 4). ER abundance also varies widely: while many genera carry few ERs on average (< 1 per genome), others are ER-rich, including *Borrelia* (8.22 ERs/genome), *Arsenophonus* (7.86), and *Pseudosulfitobacter* (7.30). Together, these patterns indicate that ERs can restructure genome content and organization either by contributing a large fraction of genomic DNA through a single large replicon or by accumulating multiple replicons.

### Large lineage-associated ERs are maintained at chromosome-level copy number

ERs can represent a substantial fraction of total genome content, so their stable maintenance is expected to require tight dosage control. We therefore asked at what copy number large lineage-associated ERs are maintained relative to the chromosome. We performed MFA on 14 strains carrying such ERs, spanning 5 taxonomic classes and 11 genera: Alphaproteobacteria (*Allorhizobium*, *Brucella*, *Cereibacter, Paracoccus, Sinorhizobium*), Betaproteobacteria (*Burkholderia, Cupriavidus*), Gammaproteobacteria (*Pseudoalteromonas, Vibrio*), Deinococci (*Deinococcus*), and Spirochaetia (*Leptospira*). MFA infers replication-related dosage from sequencing coverage. To estimate baseline copy number independently of ongoing replication, we sequenced DNA from stationary-phase cultures, where replication is expected to have ceased. Under these conditions, read coverage was nearly uniform across replicons, consistent with the absence of active replication (Associated Data 8). Two exceptions, *Leptospira biflexa* and *Deinococcus radiodurans*, showed residual replication signals despite extended cultivation under multiple conditions, likely reflecting species-specific growth dynamics. Across all strains, chromosome-to-ER coverage ratios were ~1 at both origin and terminus (ori/ori and ter/ter), consistent with large lineage-associated ERs being maintained at approximately the same copy number as the chromosome (Associated Data 10).

### Large lineage-associated ERs generally terminate replication synchronously with the chromosome

Having established chromosome-level copy number for large ERs, we next examined how their replication dynamics are coordinated with the chromosome during exponential growth.

During exponential growth, ongoing replication results in overrepresentation of sequences near the origin of replication (*ori*) and underrepresentation near the terminus (*ter*). This produces a characteristic “roof” profile for bidirectional replication, with a peak at *ori* and a symmetrical decline toward *ter*. In contrast, unidirectional replication generates a continuous linear slope from *ori* to *ter*. Comparing profiles across replicons within the same genome enables inference of relative replication initiation and termination timing and fork progression speed^24,35,36^. Assuming an identical number of copies between replicons, if one replicon has a higher *ori* coverage than another, it likely initiates replication earlier; if both replicons reach similar coverage at their *ter*, they are expected to complete replication concurrently; and if one replicon displays a steeper slope than another, this indicates slower fork progression.

**Fig. 5:**
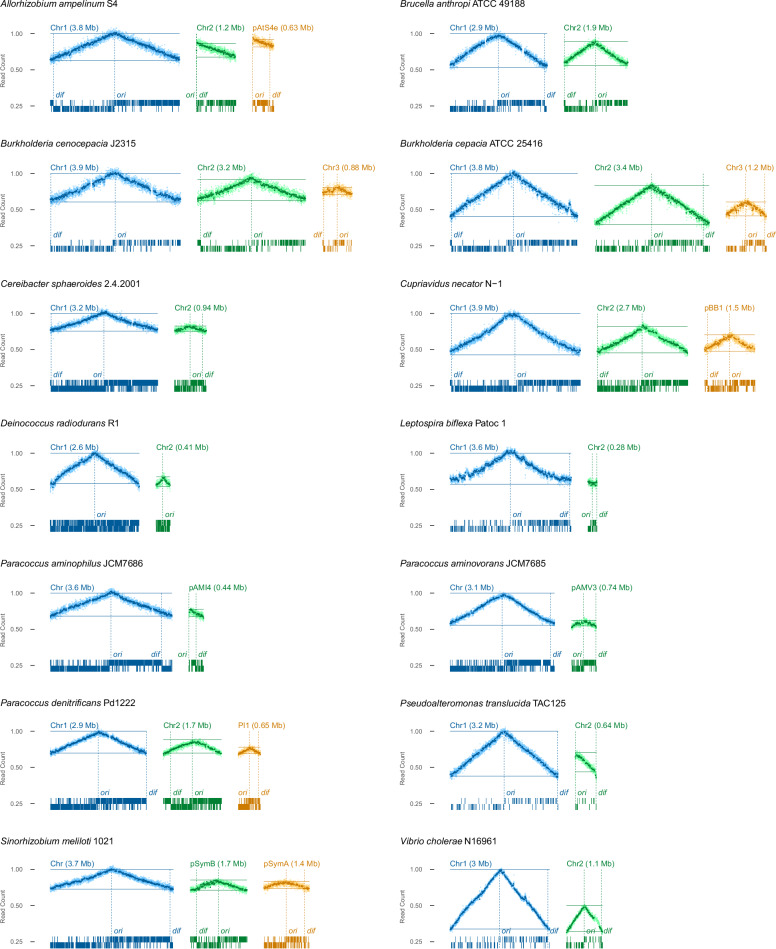
Marker frequency analysis of bacterial strains from diverse lineages containing large ERs. MFA was performed on gDNA extracted from bacteria in the exponential growth phase. The main chromosome is shown in blue, while large ERs are depicted in green and yellow. Read counts were normalized to the *ori* of the chromosome. Darker points represent normalized read counts per 10 kb windows, lighter points correspond to normalized read counts per 1 kb windows. The positions of *ori* and *dif* sites are indicated (determined as in Supplementary Data 2). Sticks represent the polar distribution of KOPS on the top and bottom strands of the respective replicons. The vertical lines indicate *ori* and *ter* read count levels (determined as in Associated Data 9). For comparison, *V. cholerae* MFA data from Val et al. (2016) were included in ref. ^24^.

Figure 5 shows that all chromosomes and most ERs exhibited bidirectional replication with clear roof profiles (Associated Data 9). A few ERs, including those in *Allorhizobium ampelinum* (Chr2, pAtS4e), *Paracoccus aminophilus* (pAMI4), and *Pseudoalteromonas translucida* (Chr2), showed a single slope consistent with unidirectional replication. Within each strain, slopes were very similar across replicons, indicating comparable fork progression between chromosome and large ERs (Associated Data 10). Across strains, the chromosome consistently showed the highest *ori* coverage, consistent with earlier initiation than large ERs. Strikingly, coverage at *ter* was very similar between chromosome and large ERs in most strains, indicating that replication termination typically occurs around the same time (Fig. 5, Associated Data 9 and Associated Data 10). A few deviations were observed, such as the third replicons in *Burkholderia* strains or pAtS4e in *A. ampelinum* S4, which appear to finish replication before the chromosome.

Overall, however, the dominant pattern across taxa is delayed initiation coupled with synchronous completion between large ERs and the chromosome, consistent with coordinated replication timing as a recurrent property of conserved large ERs.

### Large ERs show chromosome-like KOPS polarity organization around dif, consistent with FtsK-directed DNA translocation

Replication coordination is expected to be coupled to late-stage chromosome segregation^5^. In *V. cholerae* and in Enterobacteria, large ERs carry chromosome-like segregation landmarks, including polarized KOPS motifs (FtsK Orienting Polar Sequences; 5′-GGGNAGGG-3′) oriented from *ori* toward the *dif* site^31,37,38^. On chromosomes, *dif* is the XerCD recombination site used to resolve chromosome dimers prior to segregation, and FtsK is a septum-anchored DNA translocase whose oriented loading on KOPS directs translocation toward *dif*, enabling dimer resolution and faithful segregation of the terminus region^39–42^. A structural hallmark of this system is a switch in KOPS orientation at *dif*. In bacteria, *dif* is often located near the *ter* of circular chromosomes.

To test whether this architecture extends across diverse large ERs, we mapped *ori* and *dif* along each replicon and examined the distribution and orientation of KOPS motifs (Fig. 5, Associated Data 8, Associated Data 9, and Supplementary Data 2). MFA profiles indicate that dif often coincides with the point of lowest coverage (i.e., ter), but not always. This was observed in several replicons, including *Paracoccus denitrificans* Chr2, *P. aminophilus* pAMI4, and *Sinorhizobium meliloti* pSymB and pSymA, as well as in chromosomes, such as *P. aminophilus* Chr1, indicating that *dif* does not necessarily mark the site of replication termination.

Across most replicons, KOPS motifs were abundant and their polarity switch systematically occurred at *dif*, mirroring canonical chromosomal organization (Fig. 5, KOPS polarity displayed as tick marks pointing up or down depending on strand orientation). Note that in *D. radiodurans*, neither a polarized KOPS pattern nor a canonical *dif* site could be confidently identified, likely because its very high GC content (> 65%) complicates motif detection. This does not indicate the absence of terminus-region processing, as FtsK is present and functional in *D. radiodurans* and can promote Xer-dependent reactions^43^.

Overall, the conserved inversion of KOPS polarity at *dif* across large ERs supports their coupling to chromosome-like FtsK–XerCD-mediated segregation pathways.

### Hi-C reveals chromosome-like replichore alignment in large ERs

These sequence-level segregation landmarks suggest that large ERs can engage chromosome-like maintenance systems. We therefore asked whether their higher-order organization and physical proximity to the chromosome also show recurrent, structured patterns.

**Fig. 6:**
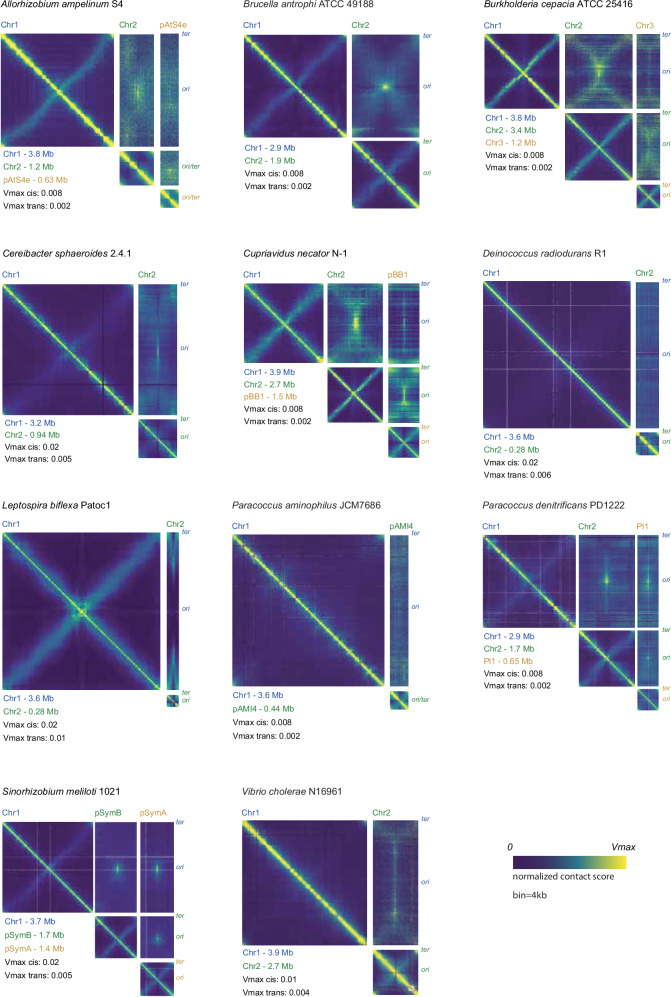
Hi-C interaction matrices between chromosome and large ERs (*ori*-centered). Normalized Hi-C contact maps (bin size = 4 kb) for eleven exponentially growing bacterial strains. For each strain, matrices are displayed with the replication origin (*ori*) centered; in some unidirectionally replicating replicons, ori co-localizes with the terminus (*ter*) and is indicated as *ori*/*ter*. Matrices with *ori* positioned at the beginning of the sequence are shown in Supplementary Fig. 1. The two-color scale indicates the normalized frequency of *cis* and *trans* contacts between genomic regions, ranging from dark blue (rare contacts) to yellow (frequent contacts). Vmax value for *cis*- and *trans*-contact are indicated under each contact map. For comparison, published Hi-C data from *V. cholerae* and *D. radiodurans* were included in refs. ^24,44^.

To assess whether large ERs physically associate with the chromosome during growth, we performed Hi-C on the MFA strain panel. After quality control, 9 Hi-C datasets were retained for analysis, representing the genera *Allorhizobium*, *Brucella*, *Burkholderia*, *Cereibacter*, *Cupriavidus*, *Leptospira*, *Paracoccus*, and *Sinorhizobium*. Hi-C datasets for *Burkholderia cenocepacia*, *Paracoccus aminovorans*, and *P. translucida* did not meet quality criteria and were excluded. Hi-C detects physical proximity between genomic loci by capturing and sequencing DNA fragments cross-linked and ligated in vivo, thereby revealing the three-dimensional organization of the genome. Hi-C can identify both intrachromosomal (*cis*) interactions and inter-replicon (*trans*) contacts, providing insight into the spatial arrangement of replicons, their interactions, and potential coordination throughout the cell cycle^23^. We performed Hi-C experiments on exponentially growing cultures. Contact maps were generated by aligning Hi-C reads to their respective reference genomes and calculating interaction frequencies across 4 kb genomic bins (Fig. 6 and Supplementary Fig. 1). For comparison, published Hi-C data from *V. cholerae* and *D. radiodurans* were included in refs. ^24,44^.

Each replicon displayed a distinct and well-defined diagonal, reflecting frequent local *cis* contacts. The larger replicons presented local domains of increased contact frequencies, separated by barriers, as already observed for all bacteria investigated so far^45^. In addition, all bidirectionally replicating replicons exhibited a more or less pronounced opposite diagonal (perpendicular to the main diagonal), reflecting interactions between the two replichores (i.e., the left and right arms of the replicon replicated outward from *ori*) (Fig. 6). This pattern likely represents the bridging of the two replichores, a process known to be mediated by cohesin-like complex SMC-ScpAB. In *Bacillus subtilis*, the SMC complex is recruited to the origin-proximal *parS* site by ParB^46,47^, tethering the two replichores together from *ori* to *ter*^48^. In our data, the replichore-bridging signal was clearly shifted in pSymB (*S. meliloti*), where the *dif* site is offset from the *ter* (Supplementary Fig. 1). In this case, the opposite diagonal is aligned with the location of *dif*, rather than with the *ter*, suggesting that SMC unloading occurs at *dif*. This is consistent with observations in *B. subtilis*, where XerD mediates SMC unloading at *dif*^49^. These findings support and generalize the idea that *dif* serves as the primary unloading site for SMC, extending this model to a broader panel of bacteria. Taken together, these Hi-C patterns indicate that large ERs adopt chromosome-like higher-order organization, including replichore alignment. Interestingly, no opposite diagonal was observed in ERs that replicate unidirectionally (Fig. 6 and Supplementary Fig. 1; *A. ampelinum* Chr2 and pAtS4e), suggesting that replichore alignment emerges specifically in bidirectionally replicating replicons and is tightly coupled to replication mode.

### *ori*-associated contacts between chromosomes and large ERs

Beyond the intra-replicon features, Hi-C revealed *trans* interactions between chromosomes and large ERs, most prominently near replication origins. In *Agrobacterium fabrum*, *ori*–*ori* contacts have been shown to be mediated by ParB and RepB (ParB-like) proteins^28,33^. Our analysis reveals that this interaction pattern is prominent in many species, including those with RepABC replicons (as in *A. fabrum*) as well as replicons carrying other types of replication systems (Fig. 6 and Supplementary Data 2). In most cases, these contacts were sharply focalized between the *oris*, as observed in *S. meliloti* (Chr/pSymB, Chr/pSymA, and pSymB/pSymA), *Cereibacter sphaeroides* (Chr1/Chr2), and *A. ampelinum* (Chr1/Chr2 and Chr2/pAtS4e) (Fig. 6). In fewer cases, one *ori* exhibited interactions with a broader region of the other replicon. For instance, in *Burkholderia cepacia or Cupriavidus necator*, the *ori* of Chr2 displayed extensive contact with a large portion of Chr1. Similar asymmetric interactions have been described in *V. cholerae*, where *ori2* on Chr2 frequently contacts the *crtS* region on the right replichore of Chr1, a site required to trigger replication initiation of Chr2^24,50^ (Fig. 6). These *ori*-associated *trans* contacts may reflect functional coordination between replicons, potentially involved in replication initiation control or origin positioning, and may point toward additional regulatory mechanisms.

### *Trans*-replichore contacts between chromosomes and large ERs

In addition to *ori*-associated interactions, we observed extended *trans* contacts between chromosomes and large ERs in several species, including *C. necator*, *A. ampelinum*, *C. sphaeroides*, and *B. cepacia* (Fig. 6 and Supplementary Fig. 1). These interactions followed an *ori*-to-*ter* axis along the replichores of both the chromosome and large ERs, suggesting extended physical proximity during replication as already observed in *V. cholerae*^24^.

In *B. cepacia*, for example, a strong *ori2* contact with Chr1 was followed by weaker but continuous interactions extending toward the *ter* regions of both Chr1 and Chr2, resulting in a characteristic cross-shaped pattern between the two replicons (Fig. 6—Chr1/Chr2 trans contact maps—right and left panels, Supplementary Fig. 1). Similar cross-shaped patterns were also evident in *C. necator* (Chr1/Chr2) and *C. sphaeroides* (Chr1/Chr2). In *L. biflexa*, exceptionally pronounced *ter*-to-*ter* contacts were observed between Chr1 and Chr2. Such trans-replichore interactions have also previously been reported in *V. cholerae*^24^, *A. fabrum*^28^, indicating that this spatial arrangement recurs across phylogenetically distant multipartite genomes and may reflect conserved replication-driven organization principles.

Notable deviations from this symmetric pattern were also informative. In *A. ampelinum*, contacts between Chr1 and Chr2 (as well as pAtS4e) followed an asymmetric, inverted-C-shaped pattern, which is most clearly visible when the Chr2 origin is set at the beginning of the sequence (Supplementary Fig. 1). This unusual topology likely reflects the unidirectional replication of Chr2 and pAtS4e, each having only a single replichore that interacts with both replichores of Chr1, which replicates bidirectionally (Fig. 5).

Together, these observations indicate that chromosome–large ER contacts are structured along replication-defined axes, and that their geometry reflects the replication mode of the interacting replicons. This supports the view that large ERs are spatially organized alongside the chromosome during replication, consistent with coupling to chromosome-centered, replication-linked maintenance programs.

## Discussion

Observations in *Vibrio* and *Agrobacterium* suggested that large ERs can show coordinated replication timing and spatial association with the chromosome, but the prevalence of these features across bacterial diversity has remained unknown. Here, using >40,000 complete genomes to map ER distributions and applying MFA and Hi-C to representative strains across multiple lineages, we find that ERs in the upper size range converge toward chromosome GC content and are maintained at ~1:1 copy number, initiate later than the chromosome, and typically complete replication synchronously, with frequent chromosome–ER contacts including *ori*-associated interactions and replichore-aligned patterns.

A central outcome of our genome-wide census is that large ERs converge in base composition toward their host chromosome. When ERs are analyzed in chromosome-normalized space, ER GC content approaches chromosomal GC content as ER size increases across essentially all well-sampled taxa (Figs. 2 and 3). This trend is largely obscured in absolute GC–size space (Fig. 1), but it becomes striking once ERs are evaluated relative to their own chromosomal background. Compositional convergence is a hallmark of long-term co-evolution, and suggests that most large ERs have been retained long enough to be shaped by host mutational and selective pressures^4^. Consistent with this view, Fig. 4 shows that large ERs are concentrated in specific genera where they recur at high prevalence and represent a substantial share of genomic DNA, supporting the view that many large ERs are lineage-associated genome components rather than transient acquisitions.

Notably, the chromosome-normalized framework also highlights a minority of large ERs that remain compositionally distinct from their host chromosome. Examples include *Pseudomonas* sp. Leaf58 pBASL58 (ΔGC = −6.9; 16.6%chr), *Pantoea* sp. BJ2 plasmindB (ΔGC = −8.4; 10.5%chr), and *Providencia* sp. PROV007 plasmid (ΔGC = +9.0; 9.0%chr) (Gammaproteobacteria, Associated Data 6). Large ERs with similarly elevated |ΔGC| relative to their host chromosome were recently reported in Enterobacteriaceae^51^. These outliers may represent a distinct class of large elements that are transient or recently acquired, and that engage in specific host interactions which may not rely on full domestication.

Our results identify a consistent set of properties shared by conserved, lineage-associated large ERs across phylogenetically distant bacteria. MFA indicates that these replicons are typically maintained at approximately chromosome-level copy number, initiate replication after the chromosome, and in most strains complete replication at similar times. Hi-C further reveals structured chromosome–ER contacts, with recurrent *ori*-associated interactions and, in several cases, extended *trans* contacts aligned along replication-defined axes. Together, these findings support the view that large, lineage-associated ERs are integrated into chromosome-centered programs coordinating replication completion, spatial organization, and terminus-region processing.

Hi-C contact maps reveal frequent *trans* contacts concentrated near the *ori* of large ERs and the chromosome (Fig. 6). Such *ori–ori* interactions were previously characterized in *A. fabrum*, where they are mediated by ParB proteins, and are thought to coordinate the positioning and segregation of *ori* regions^33^. In our dataset, a similar origin-centered interaction pattern is observed in multiple species and across different types of large replicons (not only *repABC* replicons, as it was shown for *A. fabrum*), suggesting that origin co-localization represents a common organizational strategy in multipartite genomes.

MFA data indicate that large ERs initiate replication later than the chromosome, but in most strains, terminate replication at nearly the same time. This convergence in completion timing is observed alongside similar fork progression rates across replicons within a given strain, consistent with tuning to the chromosomal replication program. Because late stages of the bacterial cell cycle impose spatial and temporal constraints on genome processing at the division site, coordinated completion may help ensure that large ERs reach a final replicated state when chromosome-linked processing and segregation activities are active and properly localized. Consistent with this view, large ERs display abundant KOPS motifs with a polarized orientation that inverts at *dif* (Fig. 5), mirroring canonical chromosomal organization. In systems where KOPS guides directional FtsK translocation toward *dif*, this architecture is consistent with engagement of FtsK–XerCD-associated terminus processing pathways. The observation that *dif* does not always coincide with the point of lowest MFA coverage (i.e., inferred *ter*) further emphasizes that *dif* is a functional landmark for late-stage processing rather than a proxy for replication termination. This interpretation aligns with prior work implicating FtsK in the segregation of large ERs such as *V. cholerae* Chr2 and the ~180 kb ER R27 in *Escherichia coli*^31,37^.

In several species, *trans* contact maps show interaction patterns consistent with prolonged proximity between chromosomes and large ERs along regions that are replicated in parallel. These “replichore-aligned” contacts suggest that chromosome and large ER replication processes may be coordinated not only in time but also in space. One possible explanation is partial physical coupling of replication processes, although alternative explanations, such as constrained positioning within the nucleoid and/or coupling to terminus-region processing and segregation, may also contribute. Notably, this link between replication and *trans*-replicon interactions is particularly evident in cases where ERs exhibit atypical replication patterns. For instance, in *A. ampelinum*, Chr2 follows a unidirectional replication mode, while in *C. sphaeroides*, Chr2 termination site is not directly opposed to its origin (Fig. 5). In these systems, inter-replicon interactions align with the progression from *ori* toward *ter* (Fig. 6 and Supplementary Fig. 1), consistent with replication dynamics shaping the interaction landscape. A similar pattern has been seen in *V. cholerae*, where *trans*-replicon interactions start between *crtS* and *ori2* and end near the *ter* regions, mirroring concurrent replication of Chr1 and Chr2^24^. This coupling could be driven by shared molecular elements, such as helicases and polymerases, as most ERs lack a complete replication system beyond the initiator. Consistent with this, in most cases, all replichores within the same genome show similar MFA slopes, indicating comparable fork speeds. These observations support the idea that coordinated replication dynamics can contribute to the spatial integration and potentially the faithful inheritance of multiple large replicons.

Together, these findings provide an experimental basis for understanding how large ERs are maintained and domesticated in multipartite genomes, and for testing the underlying mechanisms in a lineage-specific manner. Several directions follow from this work:

- Refining ER classification beyond GC–size space: Adding additional dimensions such as plasmid copy number could further refine this separation, but it is unlikely to produce a complete split between chromosomes and large ERs, because the largest ERs resemble chromosomes simultaneously in GC content, size, and copy number^11,12^. Another promising direction is to combine gene content and synteny using large language models to identify related large-ER lineages across taxa, consistent with recent work on large replicon lineages^52^. Recent studies that apply explicit genetic and functional criteria to chromid-like elements also provide a complementary route toward more mechanistic classifications^34^.
- Mechanisms coupling ER and chromosome replication: The replication patterns observed across representative strains are consistent with the existence of lineage-specific mechanisms that coordinate ER initiation with the chromosome. Identifying such “timers” in additional systems would extend what is currently known from crtS in *V. cholerae* and methylation-based control in *A. tumefaciens*^24,53^. In parallel, the recurrent replichore-aligned contact patterns raise a related mechanistic question: whether they reflect physical coupling of replication processes, or coordinated positioning that facilitates access to shared genome maintenance pathways.
- Domestication intermediates and compositional outliers: A minority of large ERs remain compositionally distinct from their host chromosome, which is consistent with more recent acquisition or incomplete domestication. These elements may offer useful entry points to study intermediate stages along domestication trajectories. Applying the same experimental framework used here, including MFA replication timing and Hi-C contacts, to such outliers could help distinguish transient elements from stably maintained, lineage-associated large ERs.
- Broadening taxonomic sampling and multipartite architectures: Although the taxonomic signal for large ERs is broadly stable across successive genome surveys, complete genomes remain unevenly distributed across bacterial diversity. It therefore remains possible that additional genera carrying conserved large ERs will be identified as sampling expands, as illustrated by recently described multipartite lineages such as *Flammeovirga* spp. and *Embleya* spp.^54,55^. More generally, considering ER abundance and total ER genomic share highlights architectures in which multiple medium-sized ERs collectively account for a substantial fraction of genomic DNA, for example, in *Azospirillum*, *Caballeronia*, and *Borrelia*. These cases motivate further work on how replication and segregation are coordinated in highly multipartite genomes.

## Methods

### ER dataset

A bacterial genome dataset was obtained from the NCBI RefSeq repository, specifically including all complete genomes available as of October 10, 2024. Genomes classified as atypical, metagenome-assembled genomes, or originating from large multi-isolate projects were excluded to ensure high-quality genome representation. To ensure data quality, several filtration steps were applied. Genomes containing replicon names labeled as “shotgun” or “partial” were removed, as these are likely incomplete assemblies, resulting in the exclusion of 60 genomes. Genomes harboring more than 30 replicons were excluded (2 genomes), a number considered implausible for truly complete bacterial genomes. Additionally, we filtered out genomes with any replicon smaller than 1 kb (55 genomes), as these may represent sequencing artifacts or incomplete assemblies. Lastly, taxonomy cleaning was performed using NCBITaxa, and the lineages that were still missing taxonomic information were removed. Following these cleaning steps, a final dataset of 43,074 genomes with 97,785 replicons was retained for analysis (Supplementary Data 1 and Associated Data 1). Note that our analysis has limitations that follow directly from the structure of available data. Because we used all complete genomes in RefSeq, some species and genera are vastly overrepresented (for example, *Escherichia coli*, *Klebsiella pneumoniae*, or *Bacillus subtilis*). We mitigate this partially by stratifying analyses across multiple taxonomic levels (Associated Data 2, Associated Data 5).

#### Chromosome and ER definition

Chromosomes were defined as the largest replicons within each genome, while all other replicons were defined as ERs. While this is a proxy definition, it is strongly supported by comparative genomic evidence: the largest replicon in bacterial genomes almost invariably harbors the DnaA-dependent origin of replication and carries the majority of essential genes^1,2,34,56^. Limitation of this approach is that it may overlook very rare and interesting cases where the main chromosome is smaller than a co-existing ER. However, such cases have never been identified in any of the closely inspected genomes^1^. This approach was used already in some bioinformatic studies and is also more practical than identifying other chromosome markers such as *dnaA* or *oriC*, which, in fact, also have their limitations, and would lead to some mismatches^34^. The *dnaA* gene can occasionally be relocated to ERs^57^ or may be absent altogether in some intracellular symbionts^56^, making it an unreliable standalone marker of chromosomal identity. Ideally, the most accurate method to identify the main chromosome would rely on locating the chromosomal origin of replication (*oriC*). However, the *oriC* sequence is not conserved across taxa, may be located in varying genetic contexts, and often requires complex comparative methods to identify. While databases such as DoriC 12.0^58^ provide automated *oriC* annotations for prokaryotic genomes, these predictions are not always accurate. In our experience, some annotated *oriC* sites do not correspond to the true replication origin (Supplementary Data 2). In summary, each chromosome identification method would result in a certain number of misannotated chromosomes. This is why we opted for the simplest method. Considering the large scale of the bioinformatic analysis, a few misannotated chromosomes and ERs do not affect the main findings of the bioinformatic portion of this study.

### Chromosome-normalized analyses of ER size and GC content

#### Statistical comparisons of chromosome and ER distributions

To quantify differences between chromosomes and ERs across taxonomic ranks, we compared the distributions of replicon size and GC content between chromosomes (largest replicon per genome) and ERs (all other replicons) within each taxon (phylum to genus). Statistical tests were applied to assess whether chromosome and ER distributions differed significantly within taxa, and results are summarized across ranks. Reports of these comparisons are provided in Associated Data 11.

#### Chromosome-normalized GC and size analyses

To compare ERs across phylogenetically diverse bacterial taxa, compositional and size metrics were expressed relative to their host chromosome. For each ER, we defined the GC difference with respect to the host chromosome as: ∆GC = GC_ER_–GC_chr_ and the relative size as: %chr = (Size_ER_/Size_chr_) × 100.

These chromosome-normalized parameters were used to evaluate compositional homogeneity and size scaling relationships independently of lineage-specific genome characteristics. Across bacterial genomes, ERs exhibited GC contents much closer to those of their host chromosomes than to chromosomes from unrelated species, indicating a strong compositional coupling between ERs and their chromosomal background (Associated Data 12). In contrast, ER size showed heterogeneous correlations with chromosome size across phyla, ranging from weakly positive to negative associations, suggesting the absence of an ER–chromosome scaling relationship (Associated Data 13).

- GC content relationships: To assess GC content relationships, we compared each ER’s GC content to that of its host chromosome and quantified this similarity as the absolute difference (|GC_ER_–GC_chr_|) (Associated Data 12). An unmatched baseline was generated by pairing each ER with chromosomes sampled from unrelated genomes. Matched and unmatched distributions were compared using a one-sided Mann–Whitney *U* test, with effect sizes estimated using Cliff’s delta. Across all genomes, ERs were markedly closer in GC content to their host chromosomes than to unrelated chromosomes (median matched GC difference was 3.0%, compared to 10.9% for unmatched pairs), a difference that was highly significant (*p* < 1 × 10⁻⁴) and associated with a large effect size (Cliff’s δ = −0.63). The same pattern was observed in every tested phylum, with matched differences consistently smaller than unmatched ones, and moderate to large effect sizes (Cliff’s δ ranging from approximately −0.30 to −0.59). These results indicate that ER GC content is strongly shaped by the compositional background of the host chromosome, supporting the use of chromosome-relative GC measures (ΔGC) for cross-taxon comparisons.
- Size relationships: To examine size relationships, we quantified the association between ER size and chromosome size using Spearman rank correlations on log10-transformed values and assessed how this relationship changed when ER size was expressed as %chr. When all data were combined, ER and chromosome sizes showed a weak positive correlation (*ρ* = +0.10). Expressing ER size as %chr shifted this association to a weak negative correlation (*ρ* = −0.13) (Associated Data 13). At the phylum level, correlations were heterogeneous. In most phyla, absolute ER–chromosome size correlations were positive but weakened after normalization (e.g., Actinomycetota +0.25 to −0.03; Cyanobacteriota +0.31 to +0.05; Campylobacterota +0.21 to +0.13). In other phyla, correlations were already negative in absolute values and became more pronounced after normalization, notably Bacteroidota (−0.15 to −0.34) and Pseudomonadota (−0.13 to −0.24). Thus, although normalization consistently shifted correlations toward lower values, lineage-level patterns remained heterogeneous, with correlations ranging from weakly positive to negative (Associated Data 13), indicating no universal ER–chromosome size scaling relationship. While the direction of this effect was consistent with our expectation that chromosome normalization would reduce lineage-driven size correlations, the absence of a common scaling pattern suggests that %chr alone is not a robust comparative metric for ER size across taxa. ER size, therefore, provides limited insight when considered in isolation but remains informative when interpreted in combination with GC content. We therefore evaluated %chr jointly with ΔGC to examine ER size and composition within a unified chromosome-relative framework.

#### 2D plotting of GC–size normalized metrics

We examined how chromosome-relative metrics affect the visual distribution of ERs in GC–size space (Associated Data 4). Normalizing GC content using ΔGC had a pronounced effect: large ERs clustered tightly around ΔGC ≈ 0, while smaller ERs remained widely dispersed, suggesting convergence toward chromosome-like base composition in large ERs. In contrast, size normalization using %chr produced only minor structural changes in the scatterplots. The impact of normalization on data structure was visualized by plotting ER distributions in four data spaces: (i) absolute GC vs. absolute size, (ii) ΔGC vs. absolute size, (iii) absolute GC vs. %chr, and (iv) ΔGC vs. %chr.

#### ER visualization and stratification by major bacterial classes

We applied the normalized metrics ΔGC and %chr across all major taxonomic ranks to visualize relationships between ERs and their host chromosomes. For every phylum, class, order, family, and genus containing at least 100 ERs, we generated interactive two-dimensional scatterplots of ΔGC versus %chr, accompanied by kernel density estimate (KDE) contours summarizing the distribution of ERs relative to their host chromosomes. Plots were produced using both linear and log₁₀ transformations of the %chr axis to facilitate comparison across replicons that differ by several orders of magnitude in size. These visualizations are provided in Associated Data 5. Building on these visualizations, finer-scale patterns were examined within major bacterial classes. For each class with at least 100 ERs, we produced ΔGC–%chr plots in which up to five genera carrying large ERs were highlighted using distinct colors, while all remaining genera were shown in grey (Figs. 2 and 3). This stratification enables direct visual comparison of ER distributions within classes and reveals how different genera contribute to the overall patterns of ER diversity. The corresponding interactive plots, available with both linear and log₁₀-scaled %chr axes, are provided in Associated Data 6. At the class level, we computed KDE distributions to identify local maxima as a function of ΔGC and %chr, as detailed in Associated Data 14.

#### Statistical assessment of ΔGC–size trends

To confirm our visual observation that large ERs tend to have ΔGC values closer to zero while small ERs show more dispersed ΔGC values, we performed a statistical analysis across all taxa containing ≥100 ERs. The analysis was carried out separately for ERs with ΔGC < 0 and ΔGC > 0. Across nearly all taxa and at every rank (phylum, class, order, family, genus), we observed a statistically significant trend toward ΔGC ≈ 0 with increasing ER size, indicating convergence toward the chromosomal GC content. Statistically significant trends in the opposite direction, i.e., divergence away from ΔGC = 0 with increasing size, were essentially absent, occurring only in a very small number of taxa on the ΔGC < 0 side (Bacteroidaceae, Campylobacteraceae, Helicobacteraceae, Neisseriaceae, Paenibacillaceae, *Staphylococcus*, *Bacteroides*, *Bradyrhizobium*) and on the ΔGC > 0 side (*Proteus*, Morganellaceae, Phyllobacteriaceae, *Phaeobacter*). All statistical models, slope estimates, significance tests, and taxon-specific results are provided in Associated Data 15.

### Taxonomic bacterial tree with ER prevalence

The genus-level tree shown in Fig. 4 was generated using the NCBI taxonomy and visualized in iTOL along with three metadata tracks^59^. All numerical values underlying Fig. 4 are available in Associated Data 16.

We restricted the analysis to genera represented by at least ten complete genomes. For each genus, we computed three ER-related metrics:

(i) % genomes with ERs: for each genome, we identified the largest ER and assigned it to a chromosome-normalized size category using 5%chr bins (e.g., 0–5%, 5–10%, …). Each genome therefore contributes to exactly one bin, defined solely by the size of its largest ER; for example, a genome whose largest ER represents 44% of chromosome length falls into the 40–45%chr bin, regardless of additional smaller ERs. For each genus, we then calculated the percentage of genomes occupying each size bin.
(ii) ER vs. chr genomic share: we quantified the proportion of total genomic content encoded on ERs, defined as the total ER bp divided by the total genome bp (chromosomes + ERs).
(iii) ER abundance per genome: we quantified the mean number of ERs per genome.

We then built a genus-level phylogenetic tree by mapping each genus name to its NCBI taxonomic identifier and retrieving the corresponding topology with ETE3/NCBITaxa. Leaf labels were formatted as “Genus N”, where N is the number of genomes available for that genus in our dataset. Finally, we exported the tree in Newick format and produced iTOL annotation files. Tree coloring was based on phylum, except for Proteobacteria, where leaves were colored by class. The Newick tree and metadata files were uploaded to iTOL to generate the final visualization.

#### Strains and growth conditions

The bacterial strains tested in this study were *A. ampelinum* S4 (formerly *Allorhizobium vitis*), *Brucella anthropi* ATCC 49188 (formerly *Ochrobactrum anthropi*), *B. cenocepacia* J2315, *B. cepacia* ATCC 25416, *C. sphaeroides* 2.4.1 (formerly *Rhodobacter sphaeroides*), *C. necator* N-1, *D. radiodurans* R1, *L. biflexa* serovar Patoc strain “Patoc 1 (Paris)”*, P. aminophilus* JCM 7686, *P. aminovorans* JCM 7685, *P. denitrificans* PD1222, *P. translucida* TAC125 (formerly *Pseudoalteromonas haloplanktis*), and *S. meliloti* 1021.

All strains, except *A. ampelinum*, *P. translucida*, *D. radiodurans* and *L. biflexa* were grown in LB Lennox medium at 30 °C. *A. ampelinum* was grown in TY medium (bacto-tryptone 5 g/L, yeast extract 3 g/L, CaCl₂·2H₂O 0.87 g/L) at 30 °C, *P. translucida* in TYP medium (bacto-tryptone 16 g/L, yeast extract 16 g/L, NaCl 20 g/L) at 20 °C, *D. radiodurans* in TSB medium (Tryptic Soy Broth) at 30 °C, and *Leptospira* spp. in EMJH medium at 30 °C.

### Marker frequency analysis

#### Preparation of bacterial samples in exponential and stationary phases

Overnight cultures were initiated from fresh streaks on solid medium by inoculating 3 mL of liquid medium and incubating for 24 h with shaking (150 rpm). To obtain exponential phase cultures, 0.1 mL of the overnight culture was transferred into 100 mL of fresh medium and incubated in a shaking water bath (180 rpm) until reaching an OD₄₅₀ of 0.2–0.4, except for *L. biflexa*, which was grown to an OD₄₂₀ of 0.2. For exponential phase samples, bacteria were harvested by centrifuging the total culture volume in two 50 mL Falcon tubes at 4 °C for 10 min at 6000 × *g*. The supernatant was discarded, and the bacterial pellets were immediately stored at −20 °C to halt replication.

For stationary phase samples, incubation of overnight cultures was continued for an additional 8 h, except for *L. biflexa*, where cultures were grown until an OD₄₂₀ of 0.6. Cultures (0.5 mL, or 50 mL for *L. biflexa*) were centrifuged in 1.5 mL Eppendorf tubes at room temperature for 1 min at 18,000 × *g*. The supernatant was removed, and the bacterial pellets were stored at −20 °C.

#### Genomic DNA extraction and sequencing

Genomic DNA was extracted from bacterial pellets obtained from both exponential and stationary phases. For all strains except *P. translucida* TAC125, the GeneMATRIX Bacterial & Yeast Genomic DNA Purification Kit (EurX) was used. DNA extraction for *P. translucida* was performed using the DNeasy Blood & Tissue Kit (Qiagen). The extracted genomic DNA was quantified using a Qubit Fluorometer (Life Technologies), yielding DNA concentrations ranging from 30 to 300 ng/μL.

DNA Sequencing. Sequencing libraries were prepared according to the Truseq DNA PCR-Free library preparation protocol (Illumina). Equimolar pools of libraries were sequenced using a HiSeq 2500 system (paired-end 100 bp) or NextSeq 500 system (paired-end 150 bp) (Illumina), to obtain 15–20 M reads per sample.

#### Sequence analysis

Sequence reads were mapped to the NCBI reference genome sequences, or to corrected assemblies in cases where rearrangements were detected (*A. ampelinum* Chr1 and Chr2, *B. anthropi* Chr1, *P. denitrificans* Chr1) (Associated Data 17), with Bowtie2 with default settings^60^ and read counts per 1 kb window were extracted from the output with a self-made Perl script. Then, a correction factor for each 1 kb window was calculated from the stationary-phase reads and was used to normalize the exponential-phase reads (as in ref. ^24^). Windows covering repeated DNA sequences were removed using the R2R scripts^22^ and manual curation. The numbers of reads per 1 kb window were then scaled to the *ori* of the chromosome and plotted with a logarithmic ordinate^22^.

MFA was used to observe replication patterns in replicons larger than 250 kb, which served as the cutoff for our analysis. In replicating bacteria (exponential phase), MFA graphs show the highest number of reads at the origin of replication and the lowest at the terminus, indicating ongoing DNA replication. Conversely, non-replicating bacteria (stationary phase) exhibit flat MFA graphs, reflecting a lack of active replication.

Stationary phase sequencing data served two purposes: (1) to normalize the exponential phase sequencing results and (2) to determine the relative copy numbers of the chromosome and secondary replicons. We only included replicons with copy numbers similar to the main chromosome in the stationary phase to make accurate conclusions about their relative replication timing during the exponential phase. For two bacterial strains, *L. biflexa* and *D. radiodurans*, the stationary phase MFA plots did not show the expected flat shape. Despite multiple attempts to cultivate them for extended periods to reach the stationary phase, we consistently observed non-flat profiles (Associated Data 8), likely due to specific growth characteristics of these organisms. Nevertheless, we present their data, as the exponential phase results strongly suggest replication synchrony between the main chromosome and their large ERs.

### Hi-C

The different bacterial strains were grown until mid-exponential phase (OD = 0.3) in 80 mL of LB. Cultures were then treated with formaldehyde at a final concentration of 3% and then incubated for 30 min under shaking at RT followed by incubation at 4 °C during another 30 min. Formaldehyde was quenched by adding 20 mL of Glycine 2.5 M followed by an incubation of 20 min under shaking. Pellets were recovered by a centrifugation of 10 min at 10,000 × *g* and at 4 °C, washed in PBS and centrifuged again using the same settings. Supernatants were discarded and pellets were frozen in dry ice and stored at −80 °C until use. Hi-C libraries were generated using the ARIMA kit (Arima Genome-Wide Hi-C Kit). Samples were first resuspended in 1 mL of sterile water and transferred to 2 mL precellys tubes containing glass beads of 0.1- and 0.5-mm diameter (Precellys – Bertin Technology). Cells were disrupted using the Precellys apparatus (Precellys Evolution) and the following program (7500 g, 6 cycles 30 s ON/30 s OFF, 4 °C). Tubes were then centrifuged for 1 min at 1000 × *g* and 700 µL of lysate was recovered and transferred to a new 1.5 mL Eppendorf tube. Tube was centrifuged for 20 min at 16,000 × *g*, 4 °C. Supernatant was carefully removed and pellet was resuspended in 45 µL of water. We then followed the ARIMA protocol and the final library was purified using AMPure beads and eluted in 130 µL of water before processing for sequencing. Hi-C genomic libraries were first sheared at a mean size of 500 bp using a Covaris S220 apparatus, processed on streptavidin beads using the Colibri kit (ThermoFisher) as previously described^61,62^. Libraries were sequenced on NextSeq apparatus. Contact maps were generated using Hicstuff (Bowtie2—very sensitive local mode—mapping quality of 30) and the different reference genomes. Contact maps were then binned at different resolutions and balanced using cooler. Plots were then displayed using different R packages^63^ (Supplementary Code). A linear scale was applied independently for *cis*- and *trans*-contact signal with a maximum value corresponding to 99% of the maximum value contained in the different contact map.

### Reporting summary

Further information on research design is available in the Nature Portfolio Reporting Summary linked to this article.

## Supplementary information

Supplementary Information

Description of Additional Supplementary Files

Supplementary Data 1

Supplementary Data 2

Supplementary Code

Reporting Summary

Transparent Peer Review file

## Data Availability

All additional data generated and analyzed in this study are provided either in the Supplementary Data files or as Associated Data available at the GitHub repository (https://meveval-ip.github.io/ER-distribution-MFA/). The sequencing data generated in this study are publicly available via the Owey platform hosted by Institut Pasteur (https://dataset.owey.io/doi/10.48802/owey.ktBVjzxN) and through the NCBI Sequence Read Archive (SRA) under BioProject accession PRJNA1440041.

## Code availability

The R script used to generate and visualize the Hi-C contact maps is provided as Supplementary Code. Hi-C data processing was performed using hicstuff (https://github.com/koszullab/hicstuff).

## References

[1] Harrison, P. W., Lower, R. P., Kim, N. K. & Young, J. P. Introducing the bacterial ‘chromid’: not a chromosome, not a plasmid. *Trends Microbiol.* **18**, 141–148 (2010). 20080407 10.1016/j.tim.2009.12.010

[2] diCenzo, G. C. & Finan, T. M. The divided bacterial genome: structure, function, and evolution. *Microbiol Mol. Biol. Rev.* **81**, e00019-17 (2017). 28794225 10.1128/MMBR.00019-17 PMC5584315

[3] Niault, T., Czarnecki, J., Lamberioux, M., Mazel, D. & Val, M. E. Cell cycle-coordinated maintenance of the Vibrio bipartite genome. *EcoSal* **11**, eesp00082022 (2023). 10.1128/ecosalplus.esp-0008-2022 PMC10729929 38277776

[4] Ostermayer, J. et al. Taming wild replicons: evolution and domestication of large extrachromosomal replicons. *Curr. Opin. Microbiol.* **88**, 102657 (2025). 40839997 10.1016/j.mib.2025.102657

[5] Reyes-Lamothe, R. & Sherratt, D. J. The bacterial cell cycle, chromosome inheritance and cell growth. *Nat. Rev. Microbiol.* **17**, 467–478 (2019). 31164753 10.1038/s41579-019-0212-7

[6] Hulter, N. et al. An evolutionary perspective on plasmid lifestyle modes. *Curr. Opin. Microbiol.* **38**, 74–80 (2017). 28538166 10.1016/j.mib.2017.05.001

[7] San Millan, A. & MacLean, R. C. Fitness costs of plasmids: a limit to plasmid transmission. *Microbiol. Spectr.* **5**, MTBP0016-2017 (2017). 10.1128/microbiolspec.mtbp-0016-2017 PMC11687550 28944751

[8] Carroll, A. C. & Wong, A. Plasmid persistence: costs, benefits, and the plasmid paradox. *Can. J. Microbiol* **64**, 293–304 (2018). 29562144 10.1139/cjm-2017-0609

[9] Rodriguez-Beltran, J., DelaFuente, J., Leon-Sampedro, R., MacLean, R. C. & San Millan, A. Beyond horizontal gene transfer: the role of plasmids in bacterial evolution. *Nat. Rev. Microbiol.* **19**, 347–359 (2021). 10.1038/s41579-020-00497-1 33469168

[10] Reyes-Lamothe, R. et al. High-copy bacterial plasmids diffuse in the nucleoid-free space, replicate stochastically and are randomly partitioned at cell division. *Nucleic Acids Res.* **42**, 1042–1051 (2014). 24137005 10.1093/nar/gkt918 PMC3902917

[11] Maddamsetti, R. et al. Scaling laws of bacterial and archaeal plasmids. *Nat. Commun.* **16**, 6023 (2025). 40603865 10.1038/s41467-025-61205-2 PMC12222811

[12] Ramiro-Martínez, P., de Quinto, I., Lanza, V. F., Gama, J. A. & Rodríguez-Beltrán, J. Universal rules govern plasmid copy number. *Nat. Commun.* **16**, 6022 (2025). 40603299 10.1038/s41467-025-61202-5 PMC12223202

[13] Baxter, J. C. & Funnell, B. E. Plasmid partition mechanisms. *Microbiol. Spectr.* **2**, PLAS-0023-2014 (2014). 10.1128/microbiolspec.PLAS-0023-2014 26104442

[14] Planchenault, C. et al. Intracellular positioning systems limit the entropic eviction of secondary replicons toward the nucleoid edges in bacterial cells. *J. Mol. Biol.* **432**, 745–761 (2020). 31931015 10.1016/j.jmb.2019.11.027

[15] Smillie, C., Garcillan-Barcia, M. P., Francia, M. V., Rocha, E. P. & de la Cruz, F. Mobility of plasmids. *Microbiol. Mol. Biol. Rev.* **74**, 434–452 (2010). 20805406 10.1128/MMBR.00020-10 PMC2937521

[16] Bahl, M. I., Hansen, L. H. & Sorensen, S. J. Persistence mechanisms of conjugative plasmids. *Methods Mol. Biol.* **532**, 73–102 (2009). 19271180 10.1007/978-1-60327-853-9_5

[17] Hall, J. P. J., Botelho, J., Cazares, A. & Baltrus, D. A. What makes a megaplasmid? *Philos. Trans. R. Soc. Lond. B Biol. Sci.* **377**, 20200472 (2022). 34839707 10.1098/rstb.2020.0472 PMC8628078

[18] Sonnenberg, C. B. & Haugen, P. The *Pseudoalteromonas* multipartite genome: distribution and expression of pangene categories, and a hypothesis for the origin and evolution of the chromid. *G3 (Bethesda)* **11**, jkab256 (2021). 34544144 10.1093/g3journal/jkab256 PMC8496264

[19] Sonnenberg, C. B., Kahlke, T. & Haugen, P. Vibrionaceae core, shell and cloud genes are non-randomly distributed on Chr 1: an hypothesis that links the genomic location of genes with their intracellular placement. *BMC Genomics* **21**, 695 (2020). 33023476 10.1186/s12864-020-07117-5 PMC7542380

[20] Dziewit, L. et al. Architecture and functions of a multipartite genome of the methylotrophic bacterium *Paracoccus aminophilus* JCM 7686, containing primary and secondary chromids. *BMC Genomics* **15**, 124 (2014). 24517536 10.1186/1471-2164-15-124 PMC3925955

[21] Petersen, J., Frank, O., Goker, M. & Pradella, S. Extrachromosomal, extraordinary and essential-the plasmids of the Roseobacter clade. *Appl Microbiol Biotechnol.* **97**, 2805–2815 (2013). 23435940 10.1007/s00253-013-4746-8

[22] Skovgaard, O., Bak, M., Lobner-Olesen, A. & Tommerup, N. Genome-wide detection of chromosomal rearrangements, indels, and mutations in circular chromosomes by short read sequencing. *Genome Res.* **21**, 1388–1393 (2011). 21555365 10.1101/gr.117416.110 PMC3149504

[23] Marbouty, M. & Koszul, R. Generation and analysis of chromosomal contact maps of bacteria. *Methods Mol. Biol.* **1624**, 75–84 (2017). 28842877 10.1007/978-1-4939-7098-8_7

[24] Val, M. E. et al. A checkpoint control orchestrates the replication of the two chromosomes of *Vibrio cholerae*. *Sci. Adv.* **2**, e1501914 (2016). 27152358 10.1126/sciadv.1501914 PMC4846446

[25] Kemter, F. S. et al. Synchronous termination of replication of the two chromosomes is an evolutionary selected feature in Vibrionaceae. *PLoS Genet.* **14**, e1007251 (2018). 29505558 10.1371/journal.pgen.1007251 PMC5854411

[26] Du, W. L. et al. Orderly replication and segregation of the four replicons of *Burkholderia cenocepacia* J2315. *PLoS Genet.* **12**, e1006172 (2016). 27428258 10.1371/journal.pgen.1006172 PMC4948915

[27] Xie, B. B. et al. Evolutionary trajectory of the replication mode of bacterial replicons. *mBio* **12**, e02745-20 (2021). 33500342 10.1128/mBio.02745-20 PMC7858055

[28] Ren, Z. et al. Conformation and dynamic interactions of the multipartite genome in *Agrobacterium tumefaciens*. *Proc. Natl. Acad. Sci. USA* **119**, e2115854119 (2022). 35101983 10.1073/pnas.2115854119 PMC8833148

[29] Romero Picazo, D. et al. Evolution of the plant-associated Pantoea was accompanied by plasmid domestication events. *Mol. Biol. Evol.* **42**, msaf273 (2025). 41159561 10.1093/molbev/msaf273 PMC12612815

[30] Niault, T. et al. Dynamic transitions of initiator binding coordinate the replication of the two chromosomes in *Vibrio cholerae*. *Nat. Commun.* **16**, 485 (2025). 39779702 10.1038/s41467-024-55598-9 PMC11711613

[31] Val, M. E. et al. FtsK-dependent dimer resolution on multiple chromosomes in the pathogen *Vibrio cholerae*. *PLoS Genet.* **4**, e1000201 (2008). 18818731 10.1371/journal.pgen.1000201 PMC2533119

[32] Espinosa, E. et al. MatP local enrichment delays segregation independently of tetramer formation and septal anchoring in *Vibrio cholerae*. *Nat. Commun.* **15**, 9893 (2024). 39543102 10.1038/s41467-024-54195-0 PMC11564523

[33] Ren, Z. et al. Centromere interactions promote the maintenance of the multipartite genome in *Agrobacterium tumefaciens*. *mBio* **13**, e0050822 (2022). 35536004 10.1128/mbio.00508-22 PMC9239152

[34] Liu, H. et al. Unexplored diversity and potential functions of extra-chromosomal elements. *mSystems* **10**, e00175–00125 (2025). 40827884 10.1128/msystems.00175-25 PMC12456017

[35] Skovgaard, O. An additional replication origin causes cell cycle specific DNA replication fork speed. *Front. Microbiol.* **16**, 1584664 (2025). 40371120 10.3389/fmicb.2025.1584664 PMC12075136

[36] Huang, D. et al. The in vivo measurement of replication fork velocity and pausing by lag-time analysis. *Nat. Commun.* **14**, 1762 (2023). 36997519 10.1038/s41467-023-37456-2 PMC10063678

[37] Fournes, F. et al. The pathway to resolve dimeric forms distinguishes plasmids from megaplasmids in Enterobacteriaceae. *Nucleic Acids Res.* **53**, gkae1300 (2025). 39797729 10.1093/nar/gkae1300 PMC11724359

[38] Bigot, S. et al. KOPS: DNA motifs that control *E. coli* chromosome segregation by orienting the FtsK translocase. * EMBO J.* **24**, 3770–3780 (2005). 16211009 10.1038/sj.emboj.7600835 PMC1276719

[39] Bigot, S., Saleh, O. A., Cornet, F., Allemand, J. F. & Barre, F. X. Oriented loading of FtsK on KOPS. *Nat. Struct. Mol. Biol.* **13**, 1026–1028 (2006). 17041597 10.1038/nsmb1159

[40] Kennedy, S. P., Chevalier, F. & Barre, F. X. Delayed activation of Xer recombination at dif by FtsK during septum assembly in *Escherichia coli*. *Mol. Microbiol.* **68**, 1018–1028 (2008). 18363794 10.1111/j.1365-2958.2008.06212.x

[41] Stouf, M., Meile, J. C. & Cornet, F. FtsK actively segregates sister chromosomes in *Escherichia coli*. *Proc. Natl. Acad. Sci. USA* **110**, 11157–11162 (2013). 23781109 10.1073/pnas.1304080110 PMC3704039

[42] Cornet, F. et al. DNA segregation in enterobacteria. *EcoSal* **11**, eesp–0038–eesp–2020 (2023). 10.1128/ecosalplus.esp-0038-2020 PMC10729935 37220081

[43] Mishra, S., Misra, H. S. & Kota, S. FtsK, a DNA motor protein, coordinates the genome segregation and early cell division processes in *Deinococcus radiodurans*. *mBio* **13**, e0174222 (2022). 36300930 10.1128/mbio.01742-22 PMC9764985

[44] Qiu, Q.-T., Zhang, C.-Y., Gao, Z.-P. & Ma, B.-G. Spatial chromosome organization and adaptation of the radiation-resistant extremophile *Deinococcus radiodurans*. *J. Biol. Chem.* **301**, 108068 (2025). 39667503 10.1016/j.jbc.2024.108068 PMC11758949

[45] Ponndara, S., Kortebi, M., Boccard, F., Bury-Mone, S. & Lioy, V. S. Principles of bacterial genome organization, a conformational point of view. *Mol. Microbiol.* **00**, 1–11 (2024). 10.1111/mmi.15290 PMC11894783 38922728

[46] Gruber, S. & Errington, J. Recruitment of condensin to replication origin regions by ParB/SpoOJ promotes chromosome segregation in *B. subtilis*. *Cell* **137**, 685–696 (2009). 19450516 10.1016/j.cell.2009.02.035

[47] Sullivan, N. L., Marquis, K. A. & Rudner, D. Z. Recruitment of SMC by ParB-parS organizes the origin region and promotes efficient chromosome segregation. *Cell* **137**, 697–707 (2009). 19450517 10.1016/j.cell.2009.04.044 PMC2892783

[48] Wang, X., Brandao, H. B., Le, T. B., Laub, M. T. & Rudner, D. Z. Bacillus subtilis SMC complexes juxtapose chromosome arms as they travel from origin to terminus. *Science* **355**, 524–527 (2017). 28154080 10.1126/science.aai8982 PMC5484144

[49] Karaboja, X. et al. XerD unloads bacterial SMC complexes at the replication terminus. *Mol. Cell* **81**, 756–766 e758 (2021). 33472056 10.1016/j.molcel.2020.12.027 PMC7897262

[50] Cockram, C., Thierry, A., Gorlas, A., Lestini, R. & Koszul, R. Euryarchaeal genomes are folded into SMC-dependent loops and domains, but lack transcription-mediated compartmentalization. *Mol. Cell* **81**, 459–472 e410 (2021). 33382984 10.1016/j.molcel.2020.12.013

[51] Guitor, A. K. et al. Megaplasmids associate with *Escherichia coli* and other *Enterobacteriaceae*. Preprint at https://www.biorxiv.org/content/10.1101/2025.09.30.679422v2.full.pdf (2025).

[52] Krakowski, K., Orlowska, M., Kaminski, K., Bartosik, D. & Dunin-Horkawicz, S. pLAST - a tool for rapid comparison and classification of bacterial plasmid sequences. Preprint at https://www.biorxiv.org/content/10.1101/2025.11.27.689987v1 (2025). 10.1093/bioinformatics/btag574 PMC13481667 42525290

[53] Martin, S. et al. DNA methylation by CcrM contributes to genome maintenance in the *Agrobacterium tumefaciens* plant pathogen. *Nucleic Acids Res.* **52**, 11519–11535 (2024). 39228370 10.1093/nar/gkae757 PMC11514494

[54] Feng, Z. et al. The second chromosome promotes the adaptation of the genus Flammeovirga to complex environments. *Microbiol. Spectr.* **9**, e00980–00921 (2021). 34878294 10.1128/Spectrum.00980-21 PMC8653839

[55] Gomez-Escribano, J. P. et al. Evidence supporting the first secondary chromosome in actinobacteria as a hallmark of the *Embleya* genus. Preprint at https://www.biorxiv.org/content/10.1101/2025.07.03.662523v1 (2025). 10.1099/mgen.0.001704 PMC13152386 42096269

[56] Mackiewicz, P., Zakrzewska-Czerwinska, J., Zawilak, A., Dudek, M. R. & Cebrat, S. Where does bacterial replication start? Rules for predicting the oriC region. *Nucleic Acids Res.* **32**, 3781–3791 (2004). 15258248 10.1093/nar/gkh699 PMC506792

[57] Poirion, O. B. & Lafay, B. Neo-formation of chromosomes in bacteria. Preprint at https://www.biorxiv.org/content/10.1101/264945v1.full (2018).

[58] Dong, M. J., Luo, H. & Gao, F. DoriC 12.0: an updated database of replication origins in both complete and draft prokaryotic genomes. *Nucleic Acids Res.* **51**, D117–D120 (2023). 36305822 10.1093/nar/gkac964 PMC9825612

[59] Letunic, I. & Bork, P. Interactive Tree of Life (iTOL) v6: recent updates to the phylogenetic tree display and annotation tool. *Nucleic Acids Res.* **52**, W78–W82 (2024). 38613393 10.1093/nar/gkae268 PMC11223838

[60] Langmead, B. & Salzberg, S. L. Fast gapped-read alignment with Bowtie 2. *Nat. Methods* **9**, 357–359 (2012). 22388286 10.1038/nmeth.1923 PMC3322381

[61] Cockram, C., Thierry, A. & Koszul, R. Generation of gene-level resolution chromosome contact maps in bacteria and archaea. *STAR Protoc.* **2**, 100512 (2021). 34027477 10.1016/j.xpro.2021.100512 PMC8121701

[62] Moreau, P. et al. Tridimensional infiltration of DNA viruses into the host genome shows preferential contact with active chromatin. *Nat. Commun.* **9**, 4268 (2018). 30323189 10.1038/s41467-018-06739-4 PMC6189100

[63] Serizay, J., Matthey-Doret, C., Bignaud, A., Baudry, L. & Koszul, R. Orchestrating chromosome conformation capture analysis with Bioconductor. *Nat. Commun.* **15**, 1072 (2024). 38316789 10.1038/s41467-024-44761-x PMC10844600

