# Supplementary material for: Large extrachromosomal replicons are widespread across bacterial lineages and show coordinated replication termination and spatial coupling with the chromosome
Source: Nat Commun. 2026 May 2;17:5962. doi: 10.1038/s41467-026-72671-7 (PMC13342612; doi:10.1038/s41467-026-72671-7)
Supplement: Supplementary file 1 — Supplementary Information [file 41467_2026_72671_MOESM1_ESM.pdf]

Supplementary Information

Large extrachromosomal replicons are widespread across bacterial lineages and show coordinated replication termination and spatial coupling with the chromosome

Jakub Czarnecki, Morgan Lamberioux, Ole Skovgaard, Amaury Bignaud, Najwa Taib, Théophile Niault, Jasmin Ostermayer, Pascale Bourhy, Julia Bos, Elvira Krakowska, Dariusz Bartosik, Romain Koszul, Martial Marbouty, Didier Mazel, Marie-Eve Val

Supplementary Figure 1. Hi-C interaction matrices between chromosome and large ERs (*ori* at position 0).
Normalized Hi-C contact maps (bin size = 4 kb) for eleven exponentially growing bacterial strains. For each strain, matrices are displayed with replication origins (*ori*) positioned at the beginning of the contact maps. In most bidirectionally replicating replicons, this representation places the replication terminus (*ter*) near the center of the map; however, this is not necessarily the case for unidirectionally replicating replicons, where ter is offset and may lie adjacent to ori (*ori/ter*). The two-color scale indicates the normalized frequency of *cis* and *trans* contacts between genomic regions, ranging from dark blue (rare contacts) to yellow (frequent contacts). Vmax value for *cis*- and *trans*-contact are indicated under each contact map. For comparison, *V. cholerae* Hi-C data from Val et al. (2016) were included [1].

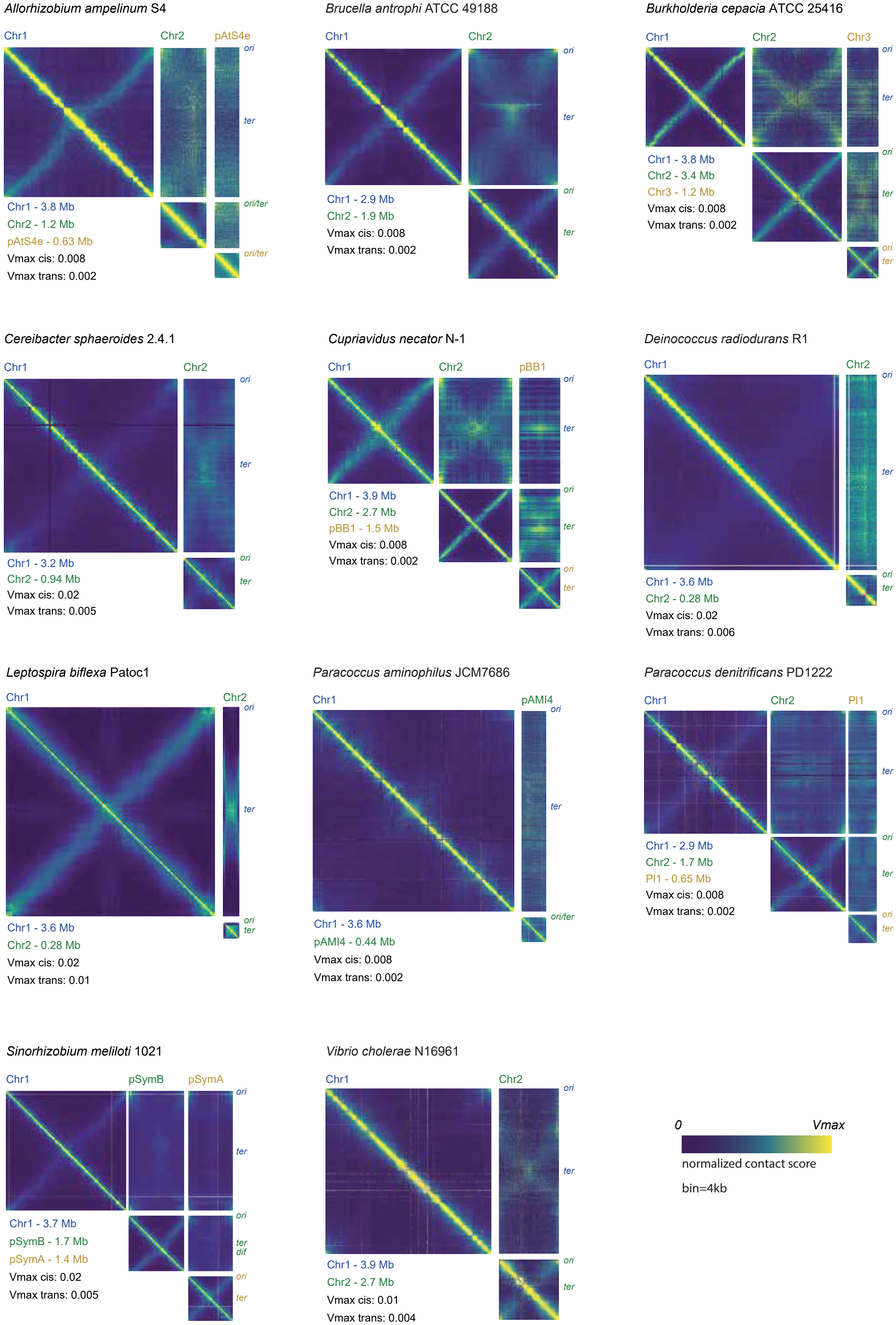
Allorhizobium ampelinum S4
Chr1
Chr2
pAtS4e
ori
ter
ori/ter
ori/ter
Chr1 - 3.8 Mb
Chr2 - 1.2 Mb
pAtS4e - 0.63 Mb
Vmax cis: 0.008
Vmax trans: 0.002
Brucella antrophi ATCC 49188
Chr1
Chr2
ori
ter
ori
ter
Chr1 - 2.9 Mb
Chr2 - 1.9 Mb
Vmax cis: 0.008
Vmax trans: 0.002
Burkholderia cepacia ATCC 25416
Chr1
Chr2
Chr3
ori
ter
ori
ter
ori
ter
Chr1 - 3.8 Mb
Chr2 - 3.4 Mb
Chr3 - 1.2 Mb
Vmax cis: 0.008
Vmax trans: 0.002
Cereibacter sphaeroides 2.4.1
Chr1
Chr2
ori
ter
ori
ter
Chr1 - 3.2 Mb
Chr2 - 0.94 Mb
Vmax cis: 0.02
Vmax trans: 0.005
Cupriavidus necator N-1
Chr1
Chr2
pBB1
ori
ter
ori
ter
ori
ter
Chr1 - 3.9 Mb
Chr2 - 2.7 Mb
pBB1 - 1.5 Mb
Vmax cis: 0.008
Vmax trans: 0.002
Deinococcus radiodurans R1
Chr1
Chr2
ori
ter
ori
ter
Chr1 - 3.6 Mb
Chr2 - 0.28 Mb
Vmax cis: 0.02
Vmax trans: 0.006
Leptospira biflexa Patoc1
Chr1
Chr2
ori
ter
ori
ter
Chr1 - 3.6 Mb
Chr2 - 0.28 Mb
Vmax cis: 0.02
Vmax trans: 0.01
Paracoccus aminophilus JCM7686
Chr1
pAMI4
ori
ter
ori/ter
Chr1 - 3.6 Mb
pAMI4 - 0.44 Mb
Vmax cis: 0.008
Vmax trans: 0.002
Paracoccus denitrificans PD1222
Chr1
Chr2
Pl1
ori
ter
ori
ter
ori
ter
Chr1 - 2.9 Mb
Chr2 - 1.7 Mb
Pl1 - 0.65 Mb
Vmax cis: 0.008
Vmax trans: 0.002
Sinorhizobium meliloti 1021
Chr1
pSymB
pSymA
ori
ter
ori
ter
dif
ori
ter
Chr1 - 3.7 Mb
pSymB - 1.7 Mb
pSymA - 1.4 Mb
Vmax cis: 0.02
Vmax trans: 0.005
Vibrio cholerae N16961
Chr1
Chr2
ori
ter
ori
ter
Chr1 - 3.9 Mb
Chr2 - 2.7 Mb
Vmax cis: 0.01
Vmax trans: 0.004
0
Vmax
normalized contact score
bin=4kb

Supplementary Table 1. Genus-level overview of largest ER prevalence and size.

Genera are summarized using arbitrary thresholds for size (>5%chr and ≥50%chr, based on the largest ER observed) and prevalence (≥60% and ≥90% of genomes carrying ERs >5%chr). Prevalence: green (≥90%); yellow (60–90%). Size: orange (≥50%chr); blue (5–50%chr).

Genus	Prevalence (% genomes with ERs >5% chr)	Size of the largest ER (%chr size)
Brucella/Ochrobactrum	98.6%	>=50% chr
Burkholderia	97.9%	>=50% chr
Cupriavidus	95.3%	>=50% chr
Azospirillum	100.0%	5-50% chr
Caballeronia	100.0%	5-50% chr
Chloracidobacterium	100.0%	5-50% chr
Leptospira	100.0%	5-50% chr
Photobacterium	100.0%	5-50% chr
Tistrella	100.0%	5-50% chr
Pseudosulfitobacter	100.0%	5-50% chr
Ralstonia	100.0%	5-50% chr
Sinorhizobium/Ensifer	100.0%	5-50% chr
Vibrio	99.5%	5-50% chr
Phaeobacter	98.3%	5-50% chr
Paraburkholderia	98.1%	5-50% chr
Rhizobium/Agrobacterium	97.4%	5-50% chr
Shinella	92.3%	5-50% chr
Pseudoalteromonas	91.5%	5-50% chr
Pantoea	87.9%	5-50% chr
Roseomonas	87.5%	5-50% chr
Deinococcus	82.1%	5-50% chr
Thermus	79.1%	5-50% chr
Prevotella	73.8%	5-50% chr
Paracoccus	73.1%	5-50% chr
Novosphingobium	72.7%	5-50% chr
Piscirickettsia	71.8%	5-50% chr
Sphingobium	69.8%	5-50% chr
Sulfitobacter	67.5%	5-50% chr
Borrelia	66.7%	5-50% chr
Komagataeibacter	64.3%	5-50% chr
Bosea	63.6%	5-50% chr
Acetobacter	62.5%	5-50% chr
Methylocystis	61.5%	5-50% chr

Supplementary Note 1. Additional description of Figure 4

ER prevalence ranged from very high in Pseudomonadota (14 192/23 681; 59.9%), Spirochaetota (238/457; 52.1%), Bacillota (4309/9916; 43.5%), Chlamydiota (92/218; 42.2%), and Cyanobacteriota (115/290; 39.7%), to moderate levels in Campylobacterota (332/1178; 28.2%), Actinomycetota (1059/4204; 25.2%), Thermodesulfobacteriota (28/127; 22.0%), Bacteroidota (300/1455; 20.6%), and Fusobacteriota (49/212; 23.1%), and finally to very low levels in Mycoplasmatota (39/704; 5.5%) and Verrucomicrobiota (3/134; 2.2%).

At finer taxonomic resolution, ER absence is common. Across the dataset, 43 genera represented by at least ten genomes contained no detectable ERs, although most of these genera were sparsely sampled. Restricting the analysis to well-sampled genera (≥50 genomes), we identified 20 genera in which ≤10% of genomes carried ERs, including *Candidatus Karelsulcia* (59 genomes, 0% ER), *Polynucleobacter* (60 genomes, 0% ER), *Bordetella* (856 genomes, 0.58% ER), *Wolbachia* (276 genomes, 0.72% ER), *Mycoplasmoides* (119 genomes, 0.84% ER), *Akkermansia* (109 genomes, 0.92% ER), *Mycoplasma* (133 genomes, 2.26% ER), *Mesomycoplasma* (90 genomes, 3.33% ER), *Mannheimia* (135 genomes, 3.70% ER), *Mycoplasmopsis* (158 genomes, 3.80% ER), *Elizabethkingia* (77 genomes, 3.90% ER), *Haemophilus* (152 genomes, 4.61% ER), *Stenotrophomonas* (125 genomes, 4.80% ER), *Treponema* (79 genomes, 5.06% ER), *Bifidobacterium* (281 genomes, 6.41% ER), *Pectobacterium* (103 genomes, 7.77% ER), *Dickeya* (71 genomes, 8.45% ER), *Streptococcus* (1,367 genomes, 8.63% ER), *Flavobacterium* (178 genomes, 8.99% ER), and *Rickettsia* (133 genomes, 9.77% ER).

Beyond prevalence, Figure 4 highlights substantial variation in ER genomic investment and ER number across genera. Most genera show minimal ER contribution (middle panel), typically <5% of total DNA on ERs even when multiple plasmids are present. A minority show high ER investment, usually driven by large replicons, including *Azospirillum* (59.4%), *Caballeronia* (57.8%), *Paraburkholderia* (51.7%), *Sinorhizobium*/*Ensifer* (44.8%), *Cupriavidus* (44.2%), *Rhizobium*/*Agrobacterium* (39.3%), *Brucella*/*Ochrobactrum* (36.8%), *Vibrio* (34.0%), and *Photobacterium* (32.2%). ER abundance per genome (right panel) also varies widely: some genera carry few ERs on average (<1), whereas others are strongly ER-rich, including *Borrelia* (8.22 ERs/genome), *Arsenophonus* (7.86), *Pseudosulfitobacter* (7.30), *Caballeronia* (5.17), and *Shinella* (5.08). More moderately ER-rich genera include *Escherichia* (2.54), *Klebsiella*/*Raoultella* (3.23), *Enterococcus* (2.83), and *Acinetobacter* (2.05).

Supplementary References

1. Val ME, et al. A checkpoint control orchestrates the replication of the two chromosomes of Vibrio cholerae. Science advances 2, e1501914 (2016).
