# Supplementary material for: Large extrachromosomal replicons are widespread across bacterial lineages and show coordinated replication termination and spatial coupling with the chromosome
Source: Nat Commun. 2026 May 2;17:5962. doi: 10.1038/s41467-026-72671-7 (PMC13342612; doi:10.1038/s41467-026-72671-7)
Supplement: Supplementary file 2 — Description of Additional Supplementary Files [file 41467_2026_72671_MOESM2_ESM.pdf]

Description of Additional Supplementary Files:

Supplementary Data 1: Replicon-level dataset of complete RefSeq bacterial genomes used in this study This table lists all replicons from the 43,074 complete RefSeq bacterial genomes analyzed (97,784 replicons total). For each replicon, it provides the assembly and RefSeq accession, organism name and taxonomic assignment (phylum→genus), replicon name, size, GC content, replicon classification (chromosome vs ER), chromosomenormalized metrics (ΔGC and %chr), the number of ERs per genome, total genome sizes, and the fraction of the genome represented by each replicon.

Supplementary Data 2: Replicon landmarks used for MFA/Hi-C analyses This table summarizes the chromosomes and ERs examined experimentally (MFA/Hi-C), including strain name, replicon identifier, RefSeq accession, inferred replication initiator type (when available; e.g., DnaA, RepABC/RepB), and the coordinates of the replication origin (ori) and chromosome dimer resolution (dif) site.
