# Supplementary material for: Large extrachromosomal replicons are widespread across bacterial lineages and show coordinated replication termination and spatial coupling with the chromosome
Source: Nat Commun. 2026 May 2;17:5962. doi: 10.1038/s41467-026-72671-7 (PMC13342612; doi:10.1038/s41467-026-72671-7)
Supplement: Supplementary file 7 — Transparent Peer Review file [file 41467_2026_72671_MOESM7_ESM.pdf]

nature portfolio

Peer Review File

Large extrachromosomal replicons are widespread across bacterial lineages and show coordinated replication termination and spatial coupling with the chromosome

Corresponding Author: Dr Marie-Eve Val

This manuscript has been previously reviewed at another journal. This document only contains information relating to versions considered at Nature Communications.

This file contains all reviewer reports in order by version, followed by all author rebuttals in order by version.

Version 0:

Reviewer comments:

Reviewer #1

(Remarks to the Author)
The authors have done a great job in the revision. The added experiments, analyses, and edits greatly improved the manuscript. I have no further concerns. Congratulations to the authors on such a nice story. I think this is a great paper and will have a strong impact on the research community.

Reviewer #3

(Remarks to the Author)
The manuscript has been improved and I have no further comments. The ('catchy') estimate of ca 10% bacterial taxa having ERs is now not in the abstract. I would try to include some kind of a quantification though (maybe restricted to large ERs?) as it may contribute to the study impact. There is a huge table currently included in the PDF - I guess that this is an error and the table will be supplied in a different format in the final version.

Open Access This Peer Review File is licensed under a Creative Commons Attribution 4.0 International License, which permits use, sharing, adaptation, distribution and reproduction in any medium or format, as long as you give appropriate credit to the original author(s) and the source, provide a link to the Creative Commons license, and indicate if changes were made.
In cases where reviewers are anonymous, credit should be given to 'Anonymous Referee' and the source.
The images or other third party material in this Peer Review File are included in the article's Creative Commons license, unless indicated otherwise in a credit line to the material. If material is not included in the article's Creative Commons license and your intended use is not permitted by statutory regulation or exceeds the permitted use, you will need to obtain permission directly from the copyright holder.
To view a copy of this license, visit https://creativecommons.org/licenses/by/4.0/

REVIEWERS' COMMENTS

Reviewer #1 (Remarks to the Author):

The authors have done a great job in the revision. The added experiments, analyses, and edits greatly improved the manuscript. I have no further concerns. Congratulations to the authors on such a nice story. I think this is a great paper and will have a strong impact on the research community.

Reviewer #3 (Remarks to the Author):

The manuscript has been improved and I have no further comments. The ('catchy') estimate of ca 10% bacterial taxa having ERs is now not in the abstract. I would try to include some kind of a quantification though (maybe restricted to large ERs?) as it may contribute to the study impact. There is a huge table currently included in the PDF- I guess that this is an error and the table will be supplied in a different format in the final version.

Response to the reviewers

We sincerely thank the reviewers for their positive assessment and constructive comments, which helped us improve the quality of the manuscript. We are pleased that the revision has addressed the concerns raised and that a consensus has been reached.

Regarding the suggestion to include a quantitative estimate of ER prevalence, we note that, following comments from other reviewers, we avoided defining “large” ERs using arbitrary cutoffs. We now provide detailed quantitative information across size ranges in Figure 4 and Supplementary Table 1, allowing readers to explore these distributions without imposing a threshold.

We also confirm that the large table appearing in the PDF was a formatting error corresponding to Supplementary Data 1 and 2. In the final version, these datasets are provided separately in an appropriate format.
